# Brain-Specific Gene Expression and Quantitative Traits Association Analysis for Mild Cognitive Impairment

**DOI:** 10.3390/biomedicines9060658

**Published:** 2021-06-08

**Authors:** Shao-Xun Yuan, Hai-Tao Li, Yu Gu, Xiao Sun

**Affiliations:** State Key Laboratory of Bioelectronics, School of Biological Science and Medical Engineering, Southeast University, Nanjing 210096, China; 230159460@seu.edu.cn (S.-X.Y.); 230169443@seu.edu.cn (H.-T.L.); 230198583@seu.edu.cn (Y.G.)

**Keywords:** subcortical structure, quantitative trait, longitudinal stably correlated, mild cognitive impairment, conversion

## Abstract

Transcriptome–wide association studies (TWAS) have identified several genes that are associated with qualitative traits. In this work, we performed TWAS using quantitative traits and predicted gene expressions in six brain subcortical structures in 286 mild cognitive impairment (MCI) samples from the Alzheimer’s Disease Neuroimaging Initiative (ADNI) cohort. The six brain subcortical structures were in the limbic region, basal ganglia region, and cerebellum region. We identified 9, 15, and 6 genes that were stably correlated longitudinally with quantitative traits in these three regions, of which 3, 8, and 6 genes have not been reported in previous Alzheimer’s disease (AD) or MCI studies. These genes are potential drug targets for the treatment of early–stage AD. Single–Nucleotide Polymorphism (SNP) analysis results indicated that cis–expression Quantitative Trait Loci (cis–eQTL) SNPs with gene expression predictive abilities may affect the expression of their corresponding genes by specific binding to transcription factors or by modulating promoter and enhancer activities. Further, baseline structure volumes and cis–eQTL SNPs from correlated genes in each region were used to predict the conversion risk of MCI patients. Our results showed that limbic volumes and cis–eQTL SNPs of correlated genes in the limbic region have effective predictive abilities.

## 1. Introduction

Alzheimer’s disease (AD) is a progressive and irreversible neurodegenerative disorder, accounting for more than 75% of all dementia events worldwide [1]. Approximately 35% of individuals over 80 years of age suffer from AD around the world [2]. Mild Cognitive Impairment (MCI) is the preclinical stage of AD and is clinically heterogeneous [3]. Genome–wide association studies (GWAS) have identified several susceptible single nucleotide polymorphisms (SNPs) for AD [4,5,6,7] and MCI [7]. However, GWAS can be used to understand which SNPs are associated with traits but cannot explain how the SNPs affect the traits. SNPs are likely to influence traits by regulating gene expression [8,9]. On the other hand, gene expression may be regulated by causal SNPs but not by the SNP with the lowest *p*-value within a linkage disequilibrium block.

Transcriptome sequencing can be used to study associations between whole transcription levels and traits in a specific tissue. Howevr, sampling for transcriptome sequencing is costly and difficult. Gusev et al. [10] proposed a new strategy, leveraging expression prediction to perform a transcriptome–wide association study (TWAS) to identify significant trait–expression associations. TWAS first fits tissue–specific models using reference data with both SNP genotype data and gene expression data available. Then, these models are used to predict gene expression in a new dataset with genotype data available. Finally, the predicted gene expression in each tissue is associated with corresponding traits. TWAS has been proved as an effective method to identify gene associations between gene expression and traits in specific tissues [11].

Several TWAS studies have identified multiple novel susceptibility genes for AD by combining Genotype–Tissue Expression Project (GTEx) gene expression models and genotype data of AD. Raj et al. [12] identified 21 genes with significant associations with AD in two cohorts, 8 of which were were novel. Hao et al. [13] combined TWAS and data from the International Genomics of Alzheimer’s Project (IGAP) cohort and identified 29 potential disease–causing genes, 21 of which were new. Jung et al. [14] combined tissue specifically predicted gene expression levels and polygenic risk score from 207 AD cases and 239 cognitively normal controls and found that the inclusion of polygenic risk score and gene expression features provided better performance in AD classification. Gerring et al. [15] performed a multi–tissue TWAS of AD and observed associated genes in brain and skin tissue.

The aim of our study was to identify genes potentially related with specific brain structure quantitative traits in MCI samples, reveal possible relationships with biological mechanisms, and use them for conversion analyses. We performed TWAS between predicted gene expression and longitudinal quantitative traits in six brain subcortical structures to identify longitudinally stable correlated genes for MCI. First, gene expression prediction models provided by GTEx [16] were used to predict gene expression in amygdala, hippocampus, accumbens area, caudate, putamen, and cerebellum using 286 MCI samples from the Alzheimer’s Disease Neuroimaging Initiative (ADNI) cohort. Second, the expression of genes in the above six structures was correlated with baseline and 12–month follow–up quantitative traits in the corresponding structures. Overlapping genes in baseline and 12–month follow–up were considered as longitudinally stable correlated genes in each structure. Third, fine–mapping analyses were performed on these longitudinally stable correlated genes and corresponding cis–eQTL SNPs to identify the potential regulation mechanisms. Finally, we further investigated the potentials of baseline quantitative traits and gene expression–determined cis–eQTL SNPs of longitudinally stable correlated genes for conversion analysis of MCI samples.

## 2. Materials and Methods

Data used in the preparation of this article were obtained from the ADNI database (adni.loni.usc.edu). ADNI was launched in 2003 as a public–private partnership, led by the Principal Investigator Michael W. Weiner, MD. The primary goal of ADNI is to test whether findings from serial magnetic resonance imaging (MRI), positron emission tomography (PET), other biological markers, and clinical and neuropsychological assessment can be combined to measure the progression of MCI and early AD.

### 2.1. Ethics Statement

We used the ADNI subject data collected from 50 clinic sites. The ADNI study was conducted according to Good Clinical Practice guidelines, US 21CFR Part 50—Protection of Human Subjects, and Part 56—Institutional Review Boards (IRBs)/Research Ethics Boards (REBs)—and pursuant to state and federal HIPAA regulations. Written informed consent was obtained from all participants after they had received a complete description before protocol–specific procedures were carried out based on the 1975 Declaration of Helsinki. IRBs were constituted according to applicable State and Federal requirements for each participating location. The protocols were submitted to appropriate Boards, and their written unconditional approval obtained and submitted to Regulatory Affairs at the Alzheimer’s disease Neuroimaging Initiative Coordinating Center (ADNICC) prior to commencement of the study. We have obtained permission to use data from ADNI, and the approval date is 25 November 2019.

### 2.2. Samples

A total of 819 samples of European ancestry were recruited by the ADNI cohort, and 757 of them were run on the Human610–Quad BeadChip (Illumina Inc., San Diego, CA, USA) for genotyping. Among these 757 samples, 286 MCI samples were MPRAGE N3–Scaled sMRI data available at both baseline and 12–month follow–up. MRI images marked with “N3” and “scaled” in the file name were downloaded from the ADNI dataset; these files underwent B1 bias field correction and N3 intensity nonuniformity correction [17]. The following information was also collected from the the ADNI dataset for 286 selected samples: gender, age, education years, Clinical Dementia Rating Sum of Boxes (CDR–SB) score, Mini–Mental State Examination (MMSE) score, Functional Assessment Questionnaire (FAQ) and Alzheimer Disease Assessment Scale scores (ADAS, version 11, 13 and Q4).

### 2.3. Genotype and Image Data Pre–Processing

PLINK 1.9 software [18] (Boston, MA, USA) was used for quality control of genotype data for 286 MCI samples. SNPs with a call rate smaller than 90%, Minor Allele Frequency (MAF) smaller than 10%, or deviations from the Hardy–Weinberg Equilibrium (5 × 10^−7^) were removed from the original genotype data. After quality control, imputation was performed using impute2 software [19]. After quality control and imputation, 28,571,732 SNPs were retained from the 286 MCI samples.

Freesurfer 6.0 software (Boston, MA, USA) was applied for automated segmentation and volume measurement of subcortical structures and total intracranial volume (ICV) for all selected MCI samples from MRI image data at baseline and 12–month follow–up. Left and right volumes from the same structure were summed. Adjustments were performed for subcortical structure volumes using gender, age, and ICV, using the following formulas:

(1) QT=a∗AGE+b∗GENDER+c∗ICV+d

(2) $$QT_{adj}=a*AGE_{mean}+b*GENDER_{mean}+c*ICV_{mean}+d+r$$

*QT* and *QT_adj_* represent raw quantitative trait volumes extracted using Freesurfer and adjusted quantitative trait volumes of a subcortical structure across the 286 MCI samples. *AGE*, *GENDER*, and *ICV* represent age, gender, and ICV of all MCI samples, while *AGE_mean_*, *GENDER_mean_*, and *ICV_mean_* represent mean age, mean gender, and mean ICV across all MCI samples; *d* represents error, while *r* represents residual. We first calculated coefficients of age (*a*), gender (*b*), and ICV (*c*) from a mixed linear regression model (Equation (1)). Then, adjusted volumes were calculated using Equation (2). Adjusted volumes of each subcortical structure were used as quantitative traits.

### 2.4. Correspondences among GTEx Models, Anatomical Regions, and Freesurfer–Defined Structures

We defined correspondences the GTEx models, anatomical regions, and freesurfer–defined structures. The PredictDB Data Repository provides 49 gene–predicted models based on GTEx data (www.gtexportal.org, accessed on 5 September 2020), of which 13 are brain–related gene expression predictive models. Freesurfer software provides 35 brain subcortical structures according to the Desikan–Killiany (DK) atlas template. In our study, 6 one–to–one corresponding gene expression predictive model–subcortical structure pairs were selected and assigned to three regions (Table 1).

### 2.5. Correlation between Predictive Gene Expression and Quantitative Traits

We utilized the PrediXcan software to predict gene expression based on the genotype data of all MCI samples. PrediXcan establishes a linear prediction model of gene expression in a dataset with both SNP genotype data and gene expression available (GTEx version 8) using a multivariate adaptive shrinkage regression (mashr) approach. Brain–specific gene expressions in 6 structures were predicted by combined prediction models and MCI genotype data. Brain–specific gene expression was determined by corresponding cis–eQTL SNPs from the LD reference files for the corresponding model in PredictDB Data Repository (http://predictdb.org/) (accessed on 5 September 2020).

We annotated the chromosomal locations of cis–eQTL SNPs in the corresponding genes using SNPnexus database [20] (accessed on 15 May 2021). Regulatory information for cis–eQTL SNPs were annotated using HaploReg database [21] (accessed on 15 May 2021) and RegulomeDB database [22] (accessed on 15 May 2021). HaploReg is a web–based tool for annotating SNPs, including chromosome number, protein binding, motif change. RegulomeDB can be used to predict whether an SNP affects transcription factor binding and gene expression. RegulomeDB provides a rank score of SNP, with a low score representing strong evidence of regulatory function. We used VARAdb database [23] to annotate the location of cis–eQTL SNPs in promoter or enhancer regions of corresponding genes (accessed on 15 May 2021). VARAdb determines promoters based on the basic gene annotation file release 33 from GENCODE (2 kb upstream of transcription start site) and determines super enhancers from 542 H3K27ac ChIP–seq samples from the human super–enhancer database [24].

Pearson correlation coefficients were used to calculate correlations between predicted gene expression and adjusted subcortical structure volumes in Table 1. The correlation matrix heatmaps were constructed using the *pheatmap* package (version 1.0.12) in R.

### 2.6. Conversion Analysis Based on Quantitative Traits and SNPs

The performances of quantitative traits and cis–eQTL SNPs were further evaluated in terms of their ability to determine the “time to progression” from MCI to AD via Kaplan–Meier analysis. For this evaluation of MCI samples in the ADNI dataset, the midpoint between the first follow–up with an AD diagnosis and the last follow–up without an AD diagnosis was considered as the conversion time point for MCI samples. The longest follow–up time was collected for samples who did not convert to AD, and these samples were regarded as non–conversion MCI samples [25]. First, quantitative trait volumes or genotypes of cis–eQTL SNPs were used as feature vectors to represent MCI samples and to calculate distances across all MCI samples through Euclidean distance. Hierarchical clustering was completed using stats package in R to cluster MCI samples into two subgroups. Then, we applied the “survfit” function in the *survival* package (version 3.2–7) in R and plotted Kaplan–Meier curves for the two subgroups. The median conversion time of MCI samples in the two subgroups was calculated; the group with a high medium time was regarded as a low–risk group, while the group with a low medium time was regarded as a high–risk group. A log rank test with a *p*-value less than 0.05 was considered statistically significant for median conversion time between risk groups [26].

## 3. Results

### 3.1. Sample Characteristics

The baseline characteristics of 286 MCI samples and their association with AD are shown in Table 2. The samples were obtained from patients with a mean (SD) age of 74.85 (6.97) years; 33.9% were female, 18.5% had less than 12 years of education. In accordance with their MCI diagnosis, the average scores of most neuropsychological tests were in the normal–to–low range. A total of 167 (58.4%) study participants converted to probable AD over a mean (SD) follow–up period of 25.05 (21.76) months. Of the 119 who did not convert, 45 had less than 36 months of follow–up data, whereas 71 were followed for more than 36 months. Three samples had only one follow–up visit.

### 3.2. Identification of Quantitative Traits–Related Genes

**Figure 1:**
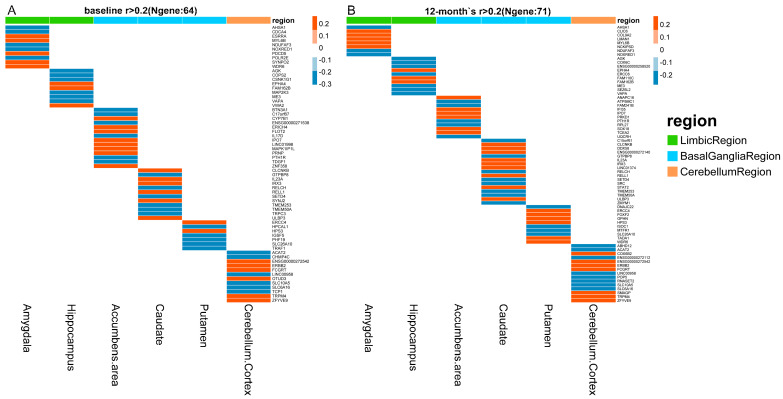
Heatmaps of correlations between predicted gene expressions and quantitative traits at baseline (**A**) and 12–month follow−up (**B**). Correlations with coefficient *r* greater than 0.2 and less than −0.2 are displayed in the heatmaps. The red color represents positive correlations, while the blue color indicates negative correlations in heatmaps. Column annotations represent brain structures for correlation analyses. For annotations, limbic region, basal ganglia region, and cerebellum region are displayed in green, sky blue, and orange, respectively.

PrediXcan software was applied to predict gene expression by integrating GTEx gene expression prediction models and ADNI genotype data. Correlations between quantitative traits and predicted gene expressions were computed by Pearson correlation across all selected samples at baseline and 12–month follow–up. The correlation heatmaps for all six structures at baseline and 12–month follow–up are shown in Figure 1. Gene–quantitative traits pairs with a correlation coefficient greater than 0.2 and lower than −0.2 are displayed in the heatmaps. Genes associated with quantitative traits were distinct across all structures at baseline (Figure 1A) and 12–month follow–up (Figure 1B). 

We evaluated the overlapping correlated genes at baseline and 12–month follow–up. Table 3 shows overlapping genes associated with structure volumes at baseline and after 12 months across all MCI samples. In the limbic region, 10 and 8 amygdala–specific expressed genes were correlated with baseline and 12–month amygdala volume, while 9 and 10 hippocampal–specific expressed genes were correlated with baseline and 12–month hippocampal volume. Four amygdala–specific expressed genes were overlapping between baseline and 12–month follow–up, while five hippocampal–specific expressed genes were overlapping between baseline and 12–month follow–up. In addition, we identified 15 overlapping genes with basal ganglia structures, including accumbens area, caudate and putamen, and 9 overlapping genes with the cerebellum. We considered these overlapping genes as stably correlated longitudinally with the corresponding quantitative traits. We used GeneCards database to annotate these genes, to define whether they were related to AD or MCI. We found that six, seven, and three genes were related to AD or MCI, while three (*NOXRED1*, *MYL6B*, and *FAM162B*), eight (*RELCH*, *IRX3*, *RELL1*, *TMEM50A*, *SETD4*, *TMEM253*, *HPS3*, *SLC26A10*), and six (*SLC6A16*, *SLC10A5*, *ENSG00000272542*, *LINC00958*, *FCGRT*, *TRPM4*) genes were potentially correlated to AD or MCI in limbic region, basal ganglia region, and cerebellum region, respectively. We summarized the potential biologic mechanisms of all these longitudinally stable correlated genes (Table S1). Genes in the limbic region are involved in energy metabolism, regulation of cell growth, apoptosis, migration and invasion, and synaptic plasticity. Genes in the basal ganglia region are involved in the inflammatory response and signal transduction. Genes in the cerebellum region are involved in signal transduction, material transport, lipid metabolism, neuronal migration, and neuritic plaques.

### 3.3. Fine-Mapping Analyses of Gene Expression-Determined Cis-eQTL SNPs

We annotated the 56 gene expression–determined cis–eQTL SNPs of all longitudinally stable correlated genes (Table 3) using SNPnexus, HaploReg, RegulomeDB, and VARAdb databases. In this study, 12, 26, and 18 SNPs were found in to 9, 15, and 9 longitudinally stable correlated genes in the limbic region, basal ganglia region, and cerebellum region, respectively. We annotated the locations of these SNPs in the corresponding genes using SNPnexus (Table S2). Among these 56 cis–eQTL SNPs, 54 SNPs (54/56, 96.4%) were in the intronic or untranslated regions of the various transcript isoforms of the genes. According to the annotation from the HaploReg database (Table S3), a total of 49 SNPs (49/56, 87.5%) can affect the corresponding genes through motifs changes, while 25 can affect the corresponding genes through proteins binding (25/56, 44.6%). According to the annotation from RegulomeDB (Table S3), 41 SNPs (41/56, 73.2%) had RegulomeDB rank scores smaller than 4, indicating transcription factor binding and location within a region of DNase hypersensitivity. We used the VARAdb database to annotate whether these cis–eQTL SNPs were located in promoters or enhancers of the corresponding genes. We found that 32 SNPs (32/56, 57.1%) were in the promoters of their corresponding genes (Table S4), while 22 SNPs were located in the forward strand, and 10 in the reverse strand. In addition, 25 SNPs (25/56, 44.6%) were enriched in super enhancers, with the corresponding genes being the closest genes (distance between the gene and the SNP was less than 1000 kb), while 13 SNPs (13/56, 23.2%) were enriched in super enhancers with the corresponding genes being the proximal genes (distance between the gene and the SNP was less than 50 kb) (Table S5). We inferred that cis–eQTL SNPs regulate the expression of the corresponding genes by affecting promoters or enhancers.

**Figure 2:**
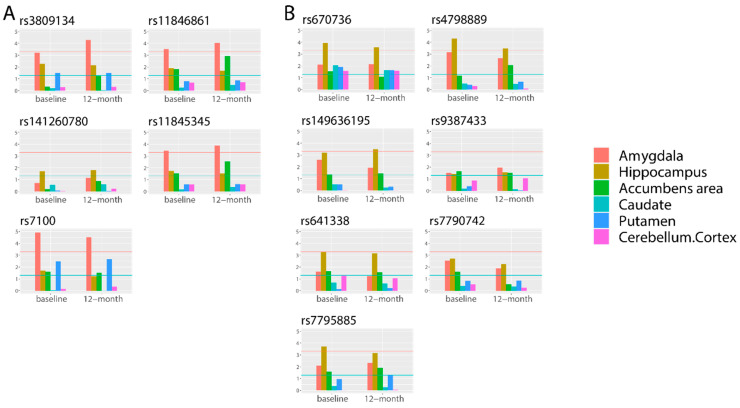
Bar plots of associations between 12 SNPs in the limbic region and 6 subcortical structures. (**A**) Five SNPs gene expression-determined SNPs in the amygdala. (**B**) Seven SNPs gene expression-determined SNPs in the hippocampus. The *X*-axis reports six subcortical structures (amygdala, hippocampus, accumbens area, caudate, putamen, and cerebellum cortex) at baseline and 12-month follow-up. The *Y*-axis presents the *p*-value (−log10) of the association based on quantitative-trait GWAS. The blue horizontal line represents −log10 (0.05), while the red horizontal line represents −log10 (5 × 10^−4^).

To evaluate whether these 56 SNPs were associated with the volume of the corresponding subcortical structures, we performed quantitative traits–based GWAS analysis using SNPs directly, instead of using predicted gene expression (Figure 2). Among five cis–eQTL SNPs for longitudinally stable correlated genes in the amygdala, four SNPs (80.0%) were significantly associated only with amygdala volume at baseline and 12–month follow–up. Among seven cis–eQTL SNPs (71.4%) for longitudinally stable correlated genes in the hippocampus, five SNPs were significantly associated only with hippocampus volume at baseline and 12–month follow–up. In the basal ganglia region and cerebellum region, 58.3% and 71.4% of SNPs were significantly associated only with corresponding quantitative traits (Figures S1 and S2). The results indicated that the correlations between quantitative traits and predicted gene expression were reasonable. On the basis of our results, we speculated that these cis–eQTL SNPs can affect both promoters and enhancers, as well as the binding of transcription factors, which may alter the expression of their target genes.

### 3.4. Conversion Analysis Based on Quantitative Traits and SNPs

**Figure 3:**
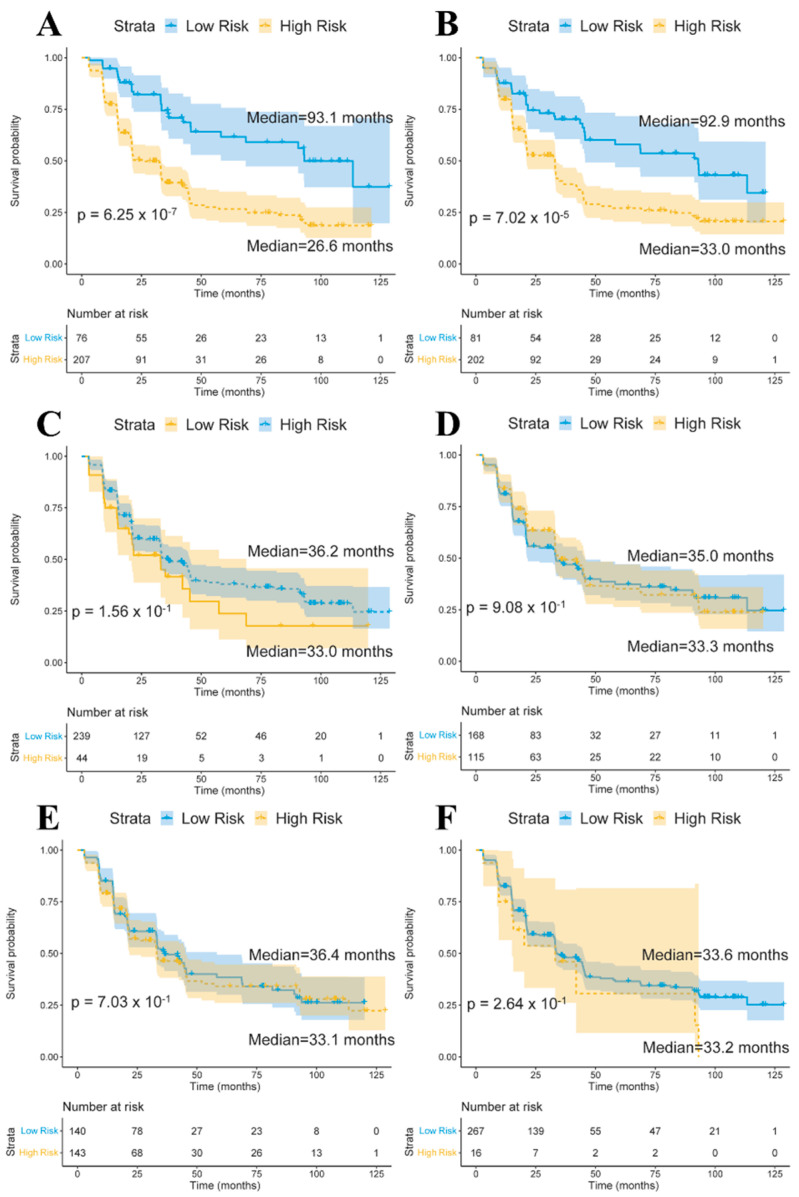
Survival curves of the two mild cognitive impairment (MCI) subgroups based on baseline volumes and cis-eQTL SNPs of limbic region (**A**,**B**), basal ganglia (**C**,**D**), cerebellum (**E**,**F**). Confidence intervals are indicated by shaded regions. The blue line represents the low-risk group, while the yellow line represents the-high risk group. Median means the median time (months) of conversion of MCI samples in the two subgroups.

We used the baseline volumes of limbic region, basal ganglia region, and cerebellum region as quantitative traits and gene expression–determined cis–eQTL SNPs of longitudinal stably correlated genes in each region to perform a conversion analysis for the MCI samples. First, the MCI samples were clustered into two subgroups using quantitative traits or SNPs. Hierarchical clustering was applied based on the Euclidean distance in the *stats* R package (v4.0.4). Then, we compared the conversion times and performed Kaplan–Meier analyses between the two MCI subgroups. Figure 3 shows the Kaplan–Meier plots for the two groups using quantitative traits and SNPs. The volumes of the structures in the limbic region and cis–eQTL SNPs of longitudinally stable correlated genes in the limbic region showed effective predictive abilities (Figure 3A,B), while this was not true for basal ganglia and cerebellum (Figure 3C–F).

**Figure 4:**
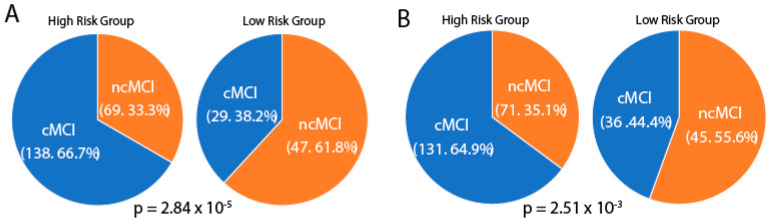
Percent of conversion mild cognitive impairment (MCI) (cMCI) and non-conversion MCI (ncMCI) samples in the high-risk group and low-risk group using quantitative traits (**A**) and SNPs derived from longitudinally stable correlated genes (**B**) in the limbic region. P, *p*-value of the chi-square test.

We calculated the percent of conversion and non–conversion of MCI samples in risk groups defined by quantitative traits and SNPs in the limbic region. Chi–square tests were used to determine between–group differences in the conversion and non–conversion of MCI samples. As shown in Figure 4, when using quantitative traits and SNPs, the high–risk groups and low–risk groups had significantly different proportions of conversion and non–conversion, with the high–risk groups showing significantly higher percentages of conversion than the low–risk groups (quantitative traits, 66.7% vs. 38.2%; SNPs: 64.9% vs. 44.4%).

## 4. Discussion

In this study, we performed transcriptome–wide association analyses between gene expressions and longitudinal quantitative traits in specific brain subcortical structures to identify longitudinally stable correlated genes for MCI. Combining gene expression prediction models generated from GTEx data and quantitative traits extracted from T1–MRI data, we identified 9, 15, and 6 genes correlated with limbic region, basal ganglia region, and cerebellum region, of which 3, 8, and 6, respectively, have not been reported in previous studies. We also performed quantitative traits–based GWAS analysis using SNPs. Most SNPs derived from previously correlated genes were directly associated with the corresponding quantitative traits, indicating that those correlations between quantitative traits and predicted gene expressions were reasonable. Furthermore, quantitative traits and gene expression–determined cis–eQTL SNPs of longitudinally stable correlated genes were used for conversion analysis of the MCI samples. We found that limbic region structure volumes and cis–eQTL SNPs derived from longitudinally stable correlated genes in the limbic region showed effective conversion predictive ability. 

Several studies performed transcriptome–wide association analyses using qualitative traits in Alzheimer’s disease. To our knowledge, this is the first research using quantitative traits in transcriptome–wide association analyses. We found that genes associated with quantitative traits of different brain structures were specific. In the limbic region, we found nine longitudinally stable correlated genes, including four for amygdala volume and five for hippocampus volume. Within these nine genes, six genes have been reported to be associated with AD or MCI based on GeneCards. For example, we found that the expression of *EPHA4* was positively correlated with hippocampus volume in baseline and 12–month follow–up. Gene expression of *EPHA4* was predicted by rs149636195 in a hippocampal predictive model. Rs149636195 is located in the 5’–untranslated region of *EPHA4* and regulates *EPHA4* expression by modulating promoter activity and enhancer activity in the hippocampus [21]. A low level of EphA4 is likely to lead to synaptic dysfunction in early AD [27], EphA4 is responsible for amyloid β–protein production regulation, and *EPHA4* mRNA levels were significantly reduced in AD brains [28]. We speculate that rs149636195 is an eQTL of *EPHA4*, and the low expression of *EPHA4* results in a decrease in hippocampal volume, which may cause synaptic dysfunction in MCI. Additionally, we identified three genes in the limbic region which have not been reported in previous AD/MCI studies, including *NOXRED1*, *MYL6B*, and *FAM162B*. *NOXRED1* (NADP–Dependent Oxidoreductase Domain–Containing 1 protein) is a key gene in oxidoreductase activity (Gene Ontology: 0016491). Oxidative stress may play a role in neuron degeneration and, thus, in AD. We suspect that *NOXRED1* may influence the pathogenesis of AD/MCI through oxidative stress. *MYL6B* encodes myosin light–chain 6B protein and is a key component of myosin. *MYL6B* contributes to memory consolidation in the amygdala [29,30]. Myosin is essential for synapse remodeling [31]. We suspect that dysregulation of *MYL6B* may affect the integrity and function of myosin, leading to the impairment of synaptic function in the pathogenesis of early–stage AD. *FAM162B* (Family with Sequence Similarity 162 Member B) is a key gene in the membrane (Gene Ontology: 0016020) and an integral component of the membrane (Gene Ontology: 0016021). *FAM162B* plays an important role in endothelial cells in the blood–brain barrier (Lifemap discovery database). We propose that *FAM162B* is important to the maintenance of the blood–brain barrier, which is required for proper synaptic and neuronal functioning. Dysregulation of *FAM162B* may cause a breakdown of the blood–brain barrier, leading to increased susceptibility to AD [32].

We investigated the potential regulation patterns of gene expression–determined cis–eQTL SNPs affecting the expression of the corresponding genes. Due to the fact that gene expression prediction models are based on fine–mapped variants that may occasionally be absent in a typical GWAS and frequently absent in older GWAS [11], we explored the annotations of SNPs for longitudinally stable correlated genes using four databases, including SNPnexus, HaploReg, RegulomeDB, and VARAdb. First, these cis–eQTL SNPs appeared to be related to specific transcription factor binding sites. Transcription factors increase or decrease the transcription levels of genes by binding to super enhancers or promoters in specific DNA regions [33]. Second, we found more that than 57% and more than 44% cis–eQTL SNPs are in the promoters and enhancers of the corresponding genes, respectively. Promoters and enhancers are responsible for the initiation and reinforcement of transcription, respectively. SNPs within enhancers can alter transcription factor binding and alter enhancer–promoter interactions, leading to dysregulation of gene expression and diseases [34], such as AD [35,36]. Based on the above observations, we inferred that gene expression–determined cis–eQTL SNPs can affect the expression of corresponding genes by altering the binding ability of some transcription factors and/or by affecting promoter and enhancer activities. We also verified the possibility of SNPs affecting corresponding gene expression. We performed association analyses using these SNPs and all quantitative traits directly. We found that most SNPs in correlated genes were also correlated to corresponding quantitative traits, indicating that the correlations between quantitative traits and gene expressions were reasonable. SNPs appeared to be associated with quantitative traits by regulating the expression of their corresponding genes.

The identified longitudinally stable correlated genes could be drug candidates for AD or MCI. *EPHA4* encodes a tyrosine protein kinase receptor, and several studies have discussed the therapeutic potential to target EphA4 for AD [37,38]. *AHSA1* encodes an activator of heat shock protein 90 (Hsp90) ATPase. Small–molecule inhibitors of Hsp90 have been successful at ameliorating amyloid beta–protein and tau protein burden in AD [39]. *MYL6B* and *VAPA* have been reported to be related to synapse formation and remodeling [40,41]. The breakdown of synaptic connections can lead to a loss of cognitive ability, and synaptic repair is a disease–modifying strategy for neurodegenerative diseases, such as AD [42]. Mitochondrial dysfunction and oxidative stress are important pathogenetic mechanism of AD [43]. Antioxidants are often used in the clinical treatment of central nervous system diseases, such as AD. Antioxidants could improve mitochondrial energy metabolism, eliminate free radicals, reduce the damage of oxidative stress to the nervous system [44]. Targeted antioxidant drugs for the treatment of AD have been developed, such as idebenone [45]. We identified four genes related to mitochondrial dysfunction and oxidative stress in the limbic region, including *NDUFAF3*, *NOXRED1*, *ME3*, and *AGK*, and these genes may be used as drug targets in early–stage AD. Meanwhile, genes in the basal ganglia region and cerebellum region are related to the inflammatory response, signal transduction, and material transport, and could also be new targets for drug development.

We investigated and compared the potential of baseline quantitative traits and cis–eQTL of longitudinally stable correlated genes in each region in predicting conversion of MCI samples. Structure volumes in the limbic region, basal ganglia region, cerebellum region and corresponding cis–eQTL SNPs in each region were used for conversion analyses. Limbic region structure volumes and 12 SNPs in from longitudinally stable correlated genes in the limbic region showed effective predictive abilities. Our results support previous MRI studies of limbic region volumes in MCI progress prediction and found that SNPs obtained by gene–quantitative trait association also showed conversion prediction value [46,47,48]. We developed an SNP panel with 12 SNPs that can be used for conversion prediction for MCI patients. Based on conversion analyses using quantitative traits and SNPs, we estimated that about 65% of MCI patients in the high–risk group will convert to AD within the established follow–up in ADNI, compared with about 40% of those in the low–risk group. 

## 5. Conclusions

In summary, our study revealed several genes which appeared to be stably correlated longitudinally with brain quantitative traits in the limbic region, basal ganglia region, and cerebellum region. These genes can be used as potential drug targets for the treatment of early–stage AD. Gene expression–determined cis–eQTL SNPs influence the expression of their corresponding genes by affecting transcription factor binding or the activities of promoters and enhancers. Quantitative traits and cis–eQTL SNPs in the limbic region can effectively predict the conversion risk of MCI patients.

## Figures and Tables

**Table 1:** Corresponence of GTEx models, anatomical regions, and subcortical structures.

GTEx Model	Region	Subcortical Structures
Brain Amygdala	Limbic	Amygdala
Brain Hippocampus		Hippocampus
Brain Caudate basal ganglia	Basal Ganglia	Caudate
Brain Putamen basal ganglia		Putamen
Brain Nucleus accumbens basal ganglia		Accumbens area
Brain Cerebellum	Cerebellum	Cerebellum cortex

GTEx models were downloaded from http://predictdb.org/ (accessed on 5 September 2020); Subcortical structures were segmented by Freesurfer software according to the Desikan–Killiany (DK) atlas template.

**Table 2:** Baseline characteristics of 286 MCI samples.

Characteristic	Number (%) or Mean ± SD
Demographic	
Age, years	74.85 ± 6.97
Gender, female	97 (33.9)
Education, ≤12 years	53 (18.5)
Neuropsychological measures	
CDRSB	1.53 ± 0.85
MMSE	27.04 ± 1.78
FAQ	3.89 ± 4.49
ADAS11	11.66 ± 4.40
ASAS13	4.40 ± 6.38
ADASQ4	18.91 ± 2.23
Conversion MCI	167 (58.4)
Conversion period	25.05 ± 21.76
Non–conversion MCI	119 (41.6)
With <3 years of follow–up data	45 (37.8)
With ≥3 years of follow–up data	71 (59.7)
With only 1 follow–up visit	3 (0.03)

MCI, Mild Cognitive Impairment; CDRSB, Clinical Dementia Rating Sum of Boxes; MMSE, Mini–Mental State Examination; FAQ, Functional Assessment Questionnaire; ADAS, Alzheimer Disease Assessment Scale scores.

**Table 3:** Overlapping quantitative traits-correlated gene sets between baseline and 12-month follow-up in six subcortical structures.

Structures	N	n	Overlap Genes	SNPs	Ranks	Annotations
Limbic Region						
Amygdala	10/8	4	*NDUFAF3* (−)	rs7100	1/1	MCI
			*NOXRED1* (−)	rs141260780 ^a^, rs11846861 ^a^	2/3	-
			*AHSA1* (−)	rs11845345 ^a^	5/4	AD/MCI
			*MYL6B* (+)	rs3809134 ^ab^	9/2	-
Hippocampus	9/10	5	*VAPA* (−)	rs4798889 ^ab^	1/5	AD/MCI
			*ME3* (−)	rs670736 ^ab^	2/1	MCI
			*AGK* (−)	rs7790742 ^a^, rs7795885 ^a^	3/9	AD/MCI
			*FAM162B* (+)	rs9387433, rs641338 ^a^	6/7	-
			*EPHA4* (+)	rs149636195 ^ab^	8/3	AD/MCI
Basal ganglia Region						
Accumbens Area	14/11	2	*PTH1R* (−)	rs2168442 ^ab^, rs144645644 ^b^	1/7	AD/MCI
			*IPO7* (+)	rs75955853 ^ab^, rs12363308 ^b^	3/1	AD
Caudate	12/17	10	*GTPBP8* (−)	rs114429530 ^ab^	1/1	AD
			*RELCH* (−)	rs3752091 ^a^, rs9958695	2/8	-
			*IRX3* (+)	rs191251428 ^ab^	4/3	-
			*CLCNKB* (+)	rs75909377 ^ab^	5/5	MCI
			*IL23A* (+)	rs79824801 ^ab^	6/10	AD/MCI
			*RELL1* (+)	rs3832308, rs4832933 ^ab^	7/7	-
			*TMEM50A* (−)	rs3093586 ^b^, rs3091243 ^b^, rs8876 ^b^	8/4	-
			*SETD4* (−)	rs2835263, rs142847892 ^a^	9/11	-
			*ULBP3* (+)	rs1537648 ^a^	10/16	AD
			*TMEM253* (−)	rs10872886	11/14	-
Putamen	7/10	3	*ERCC4* (+)	rs6498486 ^a^, rs3136042 ^a^, rs1799798 ^a^	1/1	AD/MCI
			*HPS3* (+)	rs13089410 ^a^, rs7643410 ^a^	3/4	-
			*SLC26A10* (−)	rs10747780, rs10437954	5/5	-
Cerebellum Region						
Cerebellum Cortex	12/15	9	*SLC6A16* (−)	rs8102658 ^a^	1/1	-
			*SLC10A5* (−)	rs2955002, rs58379275, rs75348453	2/2	-
			*ACAT2* (−)	rs2025187 ^ab^	3/5	AD/MCI
			*ZFYVE9* (+)	rs627011 ^ab^	4/4	MCI
			*ENSG00000272542* (+)	rs1886087, rs9518861, rs9554903	5/3	-
			*ERBB2* (+)	rs2517955 ^ab^, rs75849983 ^ab^	7/6	AD/MCI
			*LINC00958* (−)	rs111880988, rs4756736	8/15	-
			*FCGRT* (+)	rs2946865 ^ab^, rs1132990 ^b^	9/13	-
			*TRPM4* (+)	rs11882563 ^ab^, rs11083963 ^b^, rs73048855	12/9	-

N, number of correlated genes at baseline and 12-month follow-up; n, number of overlapping genes between baseline and 12-month follow-up (positive/negative correlation); Overlapping genes, overlapping genes between baseline and 12-month follow-up; SNPs, gene expression-determined cis-eQTL SNPs; Ranks, ranks of overlapping genes at baseline and 12-month follow-up; Annotations, annotations were performed using https://www.genecards.org/ (accessed on 20 March 2021). The lists of cis-eQTL SNPs of the corresponding genes were download from the LD reference file in PredictDB Data Repository (http://predictdb.org/) (accessed on 5 September 2020); SNPs with superscripts “^a^” and “^b^” indicate that these SNPs are in the promoters and enhancers of the corresponding genes, respectively.

## Data Availability

Data used in this study are available through the Alzheimer’s Disease Neuroimaging Initiative (ADNI) database (http://adni.loni.usc.edu) (accessed on 25 November 2019).

## Supplementary Materials

The following are available online at https://www.mdpi.com/article/10.3390/biomedicines9060658/s1, Table S1: Function Annotations of Selected Correlated Genes, Table S2: Genomic locations of cis-eQTL SNPs, Table S3: Annotations from HaploReg and RegulomeDB database, Table S4: Annotations of promoters of cis-eQTL SNPs, Table S5: Annotations of super enhancers of cis-eQTL SNPs, Figure S1: Bar plots of associations between 26 SNPs in the basal ganglia region and 6 subcortical structures, Figure S2: Bar plots of associations between 14 SNPs in the cerebellum region and 6 subcortical structures.

## References

[1] Van Giau V., Bagyinszky E., An S.S.A., Kim S. (2018). Clinical Genetic Strategies for Early Onset Neurodegenerative Diseases. Mol. Cell. Toxicol..

[2] Haines J.L. (2018). Alzheimer Disease: Perspectives from Epidemiology and Genetics. J. Law Med. Ethics.

[3] Hughes T.F., Snitz B.E., Ganguli M. (2011). Should Mild Cognitive Impairment Be Subtyped?. Curr. Opin. Psychiatry.

[4] Lambert J.-C., Ibrahim-Verbaas C.A., Harold D., Naj A.C., Sims R., Bellenguez C., Jun G., DeStefano A.L., Bis J.C., Beecham G.W. (2013). Meta-Analysis of 74,046 Individuals Identifies 11 New Susceptibility Loci for Alzheimer’s Disease. Nat. Genet..

[5] Jansen I.E., Savage J.E., Watanabe K., Bryois J., Williams D.M., Steinberg S., Sealock J., Karlsson I.K., Hägg S., Athanasiu L. (2019). Genome-Wide Meta-Analysis Identifies New Loci and Functional Pathways Influencing Alzheimer’s Disease Risk. Nat. Genet..

[6] Schwartzentruber J., Cooper S., Liu J.Z., Barrio-Hernandez I., Bello E., Kumasaka N., Young A.M., Franklin R.J., Johnson T., Estrada K. (2021). Genome-Wide Meta-Analysis, Fine-Mapping and Integrative Prioritization Implicate New Alzheimer’s Disease Risk Genes. Nat. Genet..

[7] Shen L., Kim S., Risacher S.L., Nho K., Swaminathan S., West J.D., Foroud T., Pankratz N., Moore J.H., Sloan C.D. (2010). Whole Genome Association Study of Brain-Wide Imaging Phenotypes for Identifying Quantitative Trait Loci in MCI and AD: A Study of the ADNI Cohort. NeuroImage.

[8] Albert F.W., Kruglyak L. (2015). The Role of Regulatory Variation in Complex Traits and Disease. Nat. Rev. Genet..

[9] Lappalainen T., Sammeth M., Friedländer M.R., AC‘t Hoen P., Monlong J., Rivas M.A., Gonzalez-Porta M., Kurbatova N., Griebel T., Ferreira P.G. (2013). Transcriptome and Genome Sequencing Uncovers Functional Variation in Humans. Nature.

[10] Gusev A., Ko A., Shi H., Bhatia G., Chung W., Penninx B.W., Jansen R., De Geus E.J., Boomsma D.I., Wright F.A. (2016). Integrative Approaches for Large-Scale Transcriptome-Wide Association Studies. Nat. Genet..

[11] Gamazon E.R., Wheeler H.E., Shah K.P., Mozaffari S.V., Aquino-Michaels K., Carroll R.J., Eyler A.E., Denny J.C., Nicolae D.L., Cox N.J. (2015). A Gene-Based Association Method for Mapping Traits Using Reference Transcriptome Data. Nat. Genet..

[12] Raj T., Li Y.I., Wong G., Humphrey J., Wang M., Ramdhani S., Wang Y.-C., Ng B., Gupta I., Haroutunian V. (2018). Integrative Transcriptome Analyses of the Aging Brain Implicate Altered Splicing in Alzheimer’s Disease Susceptibility. Nat. Genet..

[13] Hao S., Wang R., Zhang Y., Zhan H. (2019). Prediction of Alzheimer’s Disease-Associated Genes by Integration of GWAS Summary Data and Expression Data. Front. Genet..

[14] Jung S.-H., Nho K., Kim D., Won H.-H., Initiative A.D.N. (2020). Genetic Risk Prediction of Late-Onset Alzheimer’s Disease Based on Tissue-Specific Transcriptomic Analysis and Polygenic Risk Scores: Genetics/Genetic Factors of Alzheimer’s Disease. Alzheimer’s Dement..

[15] Gerring Z.F., Lupton M.K., Edey D., Gamazon E.R., Derks E.M. (2020). An Analysis of Genetically Regulated Gene Expression across Multiple Tissues Implicates Novel Gene Candidates in Alzheimer’s Disease. Alzheimer’s Res. Ther..

[16] GTEx Consortium (2017). Genetic Effects on Gene Expression across Human Tissues. Nature.

[17] Boyes R.G., Gunter J.L., Frost C., Janke A.L., Yeatman T., Hill D.L., Bernstein M.A., Thompson P.M., Weiner M.W., Schuff N. (2008). Intensity Non-Uniformity Correction Using N3 on 3-T Scanners with Multichannel Phased Array Coils. Neuroimage.

[18] Purcell S., Neale B., Todd-Brown K., Thomas L., Ferreira M.A., Bender D., Maller J., Sklar P., de Bakker P.I., Daly M.J. (2007). PLINK: A Tool Set for Whole-Genome Association and Population-Based Linkage Analyses. Am. J. Hum. Genet..

[19] Howie B.N., Donnelly P., Marchini J. (2009). A Flexible and Accurate Genotype Imputation Method for the next Generation of Genome-Wide Association Studies. PLoS Genet..

[20] Dayem Ullah A.Z., Lemoine N.R., Chelala C. (2012). SNPnexus: A Web Server for Functional Annotation of Novel and Publicly Known Genetic Variants (2012 Update). Nucleic Acids Res..

[21] Ward L.D., Kellis M. (2012). HaploReg: A Resource for Exploring Chromatin States, Conservation, and Regulatory Motif Alterations within Sets of Genetically Linked Variants. Nucleic Acids Res..

[22] Boyle A.P., Hong E.L., Hariharan M., Cheng Y., Schaub M.A., Kasowski M., Karczewski K.J., Park J., Hitz B.C., Weng S. (2012). Annotation of Functional Variation in Personal Genomes Using RegulomeDB. Genome Res..

[23] Pan Q., Liu Y.-J., Bai X.-F., Han X.-L., Jiang Y., Ai B., Shi S.-S., Wang F., Xu M.-C., Wang Y.-Z. (2021). VARAdb: A Comprehensive Variation Annotation Database for Human. Nucleic Acids Res..

[24] Jiang Y., Qian F., Bai X., Liu Y., Wang Q., Ai B., Han X., Shi S., Zhang J., Li X. (2019). SEdb: A Comprehensive Human Super-Enhancer Database. Nucleic Acids Res..

[25] Barnes D.E., Cenzer I.S., Yaffe K., Ritchie C.S., Lee S.J., Alzheimer’s Disease Neuroimaging Initiative (2014). A Point-Based Tool to Predict Conversion from Mild Cognitive Impairment to Probable Alzheimer’s Disease. Alzheimer’s Dement..

[26] Liu G.-M., Zeng H.-D., Zhang C.-Y., Xu J.-W. (2019). Identification of a Six-Gene Signature Predicting Overall Survival for Hepatocellular Carcinoma. Cancer Cell Int..

[27] Rosenberger A.F., Rozemuller A.J., van der Flier W.M., Scheltens P., van der Vies S.M., Hoozemans J.J. (2014). Altered Distribution of the EphA4 Kinase in Hippocampal Brain Tissue of Patients with Alzheimer’s Disease Correlates with Pathology. Acta Neuropathol. Commun..

[28] Tamura K., Chiu Y.-W., Shiohara A., Hori Y., Tomita T. (2020). EphA4 Regulates Aβ Production via BACE1 Expression in Neurons. FASEB J..

[29] Gavin C.F., Rubio M.D., Young E., Miller C., Rumbaugh G. (2012). Myosin II Motor Activity in the Lateral Amygdala Is Required for Fear Memory Consolidation. Learn. Mem..

[30] Lamprecht R., Margulies D., Farb C., Hou M., Johnson L., LeDoux J. (2006). Myosin Light Chain Kinase Regulates Synaptic Plasticity and Fear Learning in the Lateral Amygdala. Neuroscience.

[31] Kneussel M., Wagner W. (2013). Myosin Motors at Neuronal Synapses: Drivers of Membrane Transport and Actin Dynamics. Nat. Rev. Neurosci..

[32] Ishii M., Iadecola C. (2020). Risk Factor for Alzheimer’s Disease Breaks the Blood–Brain Barrier.

[33] Gill G. (2001). Regulation of the Initiation of Eukaryotic Transcription. Essays Biochem..

[34] Khurana E., Fu Y., Chakravarty D., Demichelis F., Rubin M.A., Gerstein M. (2016). Role of Non-Coding Sequence Variants in Cancer. Nat. Rev. Genet..

[35] Kikuchi M., Hara N., Hasegawa M., Miyashita A., Kuwano R., Ikeuchi T., Nakaya A. (2019). Enhancer Variants Associated with Alzheimer’s Disease Affect Gene Expression via Chromatin Looping. BMC Med. Genom..

[36] Choi K.Y., Lee J.J., Gunasekaran T.I., Kang S., Lee W., Jeong J., Lim H.J., Zhang X., Zhu C., Won S.-Y. (2019). *APOE* Promoter Polymorphism-219T/G Is an Effect Modifier of the Influence of APOE Ε4 on Alzheimer’s Disease Risk in a Multiracial Sample. J. Clin. Med..

[37] Fu A.K., Hung K.-W., Huang H., Gu S., Shen Y., Cheng E.Y., Ip F.C., Huang X., Fu W.-Y., Ip N.Y. (2014). Blockade of EphA4 Signaling Ameliorates Hippocampal Synaptic Dysfunctions in Mouse Models of Alzheimer’s Disease. Proc. Natl. Acad. Sci. USA.

[38] Vargas L., Cerpa W., Muñoz F., Zanlungo S., Alvarez A. (2018). Amyloid-β Oligomers Synaptotoxicity: The Emerging Role of EphA4/c-Abl Signaling in Alzheimer’s Disease. Biochim. Biophys. Acta Mol. Basis Dis..

[39] Blair L.J., Sabbagh J.J., Dickey C.A. (2014). Targeting Hsp90 and Its Co-Chaperones to Treat Alzheimer’s Disease. Expert Opin. Ther. Targets.

[40] Rudolf R., Bittins C.M., Gerdes H.-H. (2011). The Role of Myosin V in Exocytosis and Synaptic Plasticity. J. Neurochem..

[41] Matteoli M., Coco S., Schenk U., Verderio C. (2004). Vesicle Turnover in Developing Neurons: How to Build a Presynaptic Terminal. Trends Cell Biol..

[42] Lu B., Nagappan G., Guan X., Nathan P.J., Wren P. (2013). *BDNF*-Based Synaptic Repair as a Disease-Modifying Strategy for Neurodegenerative Diseases. Nat. Rev. Neurosci..

[43] Butterfield D.A., Halliwell B. (2019). Oxidative Stress, Dysfunctional Glucose Metabolism and Alzheimer Disease. Nat. Rev. Neurosci..

[44] Rottkamp C.A., Nunomura A., Raina A.K., Sayre L.M., Perry G., Smith M.A. (2000). Oxidative Stress, Antioxidants, and Alzheimer Disease. Alzheimer Dis. Assoc. Disord..

[45] Parkinson M.H., Schulz J.B., Giunti P. (2013). Co-Enzyme Q10 and Idebenone Use in Friedreich’s Ataxia. J. Neurochem..

[46] Qian L., Liu R., Qin R., Zhao H., Xu Y. (2018). The Associated Volumes of Sub-Cortical Structures and Cognitive Domain in Patients of Mild Cognitive Impairment. J. Clin. Neurosci..

[47] Xu L., Wu X., Li R., Chen K., Long Z., Zhang J., Guo X., Yao L. (2016). Prediction of Progressive Mild Cognitive Impairment by Multi-Modal Neuroimaging Biomarkers. J. Alzheimer’s Dis..

[48] Yi H.-A., Möller C., Dieleman N., Bouwman F.H., Barkhof F., Scheltens P., van der Flier W.M., Vrenken H. (2016). Relation between Subcortical Grey Matter Atrophy and Conversion from Mild Cognitive Impairment to Alzheimer’s Disease. J. Neurol. Neurosurg. Psychiatry.

