# Supplementary material for: Brain-Specific Gene Expression and Quantitative Traits Association Analysis for Mild Cognitive Impairment
Source: Biomedicines. 2021 Jun 8;9(6):658. doi: 10.3390/biomedicines9060658 (PMC8229744; doi:10.3390/biomedicines9060658)
Supplement: Supplementary file 1 [file biomedicines-09-00658-s001.zip › supplementary.pdf]

Table S1: Function ARegion StructureGene Chromosome Gene Type SNP BP Location

Region	Structures	Overlap genes	Annotations	Potential mechanism
Limbic Region	Amygdala	NDUFAF3	MCI	assembly of mitochondrial complex
		NOXRED1	-	NADP-dependent oxidoreductase
		AHSA1	AD/MCI	cell growth, apoptosis and migration
		MYL6B	-	cell adhesion, cell migration and tissue architecture, cargo transport and endocytosis
	Hippocampus	VAPA	AD/MCI	vesicular neurotransmission, membrane fusion, protein complex assembly and cell motility
		ME3	MCI	cell growth and cellular senescence
		AGK	AD/MCI	function in the mitochondria
		FAM162B	-	apoptosis
		EPHA4	AD/MCI	synaptic plasticity
Basal ganglia Region	Accumbens.area	PTH1R	AD/MCI	regulate blood calcium homeostasis
		IPO7	AD	autonomous nuclear transport receptor or as an adapter-like protein
	Caudate	GTPBP8	AD	mitochondrial translation and mitochondrial bioenergetics
		RELCH	-	intracellular cholesterol distribution
		IRX3	-	neural development
		CLCNKB	MCI	cell volume, membrane potential stabilization, signal transduction and transepithelial transport
		IL23A	AD/MCI	immune inflammatory
		RELL1	-	inflammatory response
		TMEM50A	-	unknown
		SETD4	-	methyltransferase activity
		ULBP3	AD	peptide antigen binding and natural killer cell lectin-like receptor binding
		TMEM253	-	unknown
	Putamen	ERCC4	AD/MCI	DNA repair
		HPS3	-	biogenesis Of Lysosomal Organelles
		SLC26A10	-	unknown
Cerebellum Region	Cerebellum.Cortex	SLC6A16	-	neurotransmitter transporter
		SLC10A5	-	solute carrier
		ACAT2	AD/MCI	lipid metabolism
		ZFYVE9	MCI	neuronal migration
		ENSG00000272542	-	unknown
		ERBB2	AD/MCI	neuritic plaques
		LINC00958	-	unknown
		FCGRT	-	mediates the asymmetric transcytosis of IgG across the blood–brain barrier
		TRPM4	-	calcium-activated nonselective ion channel

Table S2: Genomic locations of cis-eQTL SNPs,

Region	Structure	Gene	Chromosome	Gene Type	SNP	BP	Location
LimbicRegion	Amygdala	NDUFAF3	chr3	protein_coding	rs7100	49053219	3downstream,3utr
		NOXRED1	chr14	protein_coding	rs141260780	77890314	5upstream
		NOXRED1	chr14	protein_coding	rs11846861	77889546	intronic,5upstream
		AHSA1	chr14	protein_coding	rs11845345	77923783	5utr,intronic,non-coding intronic
		MYL6B	chr12	antisense	rs3809134	56546011	non-coding intronic
	Hippocampus	VAPA	chr18	protein_coding	rs4798889	9913930	3downstream
		ME3	chr11	protein_coding	rs670736	86383679	5upstream
		AGK	chr7	antisense	rs7790742	141250685	non-coding intronic
		AGK	chr7	antisense	rs7795885	141251044	5upstream,non-coding
		FAM162B	chr6	protein_coding	rs9387433	117077450	intronic

		FAM162B	chr6	protein_coding	rs641338	117086265	intronic
		EPHA4	chr2	protein_coding	rs149636195	222438911	5utr,5upstream
BasalGangliaRegion	AccumbensArea	PTH1R	chr3	protein_coding	rs2168442	46919379	intronic
		PTH1R	chr3	protein_coding	rs144645644	46934290	intronic,non-coding
		IPO7	chr11	protein_coding	rs75955853	9406129	3downstream
		IPO7	chr11	protein_coding	rs12363308	9409801	intronic
	Caudate	GTPBP8	chr3	protein_coding	rs114429530	112710133	coding nonsyn,5upstream
		RELCH	chr18	protein_coding	rs3752091	59854253	5upstream,intronic,5utr,non-coding
		RELCH	chr18	protein_coding	rs9958695	59858801	intronic
		IRX3	chr16	antisense	rs191251428	54320835	non-coding intronic
		CLCNKB	chr1	protein_coding	rs75909377	16370712	intronic
		IL23A	chr12	protein_coding	rs79824801	56728137	non-coding intronic
		RELL1	chr4	protein_coding	rs4832933	37688443	5upstream
		TMEM50A	chr1	protein_coding	rs3093586	25668826	5upstream,non-coding intronic,intronic
		TMEM50A	chr1	protein_coding	rs3091243	25669057	5upstream,non-coding intronic,intronic
		TMEM50A	chr1	protein_coding	rs8876	25687308	3utr,non-coding
		SETD4	chr21	protein_coding	rs2835263	37430245	5upstream,non-coding intronic,intronic
		SETD4	chr21	protein_coding	rs142847892	37451298	intronic
	Putamen	ULBP3	chr6	protein_coding	rs1537648	150389924	intronic
		TMEM253	chr14	None	rs10872886	21574690	None
		ERCC4	chr16	protein_coding	rs6498486	14013666	3downstream
		ERCC4	chr16	protein_coding	rs3136042	14014055	coding syn|syn,non-coding
		ERCC4	chr16	protein_coding	rs1799798	14014278	intronic,non-coding intronic
		HPS3	chr3	protein_coding	rs13089410	148847441	non-coding,5utr,5upstream
		HPS3	chr3	protein_coding	rs7643410	148848073	non-coding intronic,intronic,non-coding
		SLC26A10	chr12	None	rs10747780	57980541	None
		SLC26A10	chr12	None	rs10437954	58003922	None
CerebellumRegion	Cerebellum	SLC6A16	chr19	protein_coding	rs8102658	49828855	5upstream
		SLC10A5	chr8	TEC	rs2955002	82608878	non-coding
		SLC10A5	chr8	TEC	rs58379275	82609783	non-coding
		SLC10A5	chr8	protein_coding	rs75348453	82626146	intronic,non-coding intronic,3downstream
		ACAT2	chr6	protein_coding	rs2025187	160181435	intronic
		ZFYVE9	chr1	protein_coding	rs627011	52608109	non-coding intronic,intronic
		ENSG00000272542	chr13	transcribed_unprocessed_pseudogene	rs1886087	103535828	non-coding intronic
		ENSG00000272542	chr13	transcribed_unprocessed_pseudogene	rs9518861	103541616	5upstream,non-coding intronic
		ENSG00000272542	chr13	transcribed_unprocessed_pseudogene	rs9554903	103542220	non-coding intronic,5upstream
		ERBB2	chr17	protein_coding	rs2517955	37843681	non-coding intronic,intronic
		ERBB2	chr17	protein_coding	rs75849983	37843762	non-coding intronic,intronic
		LINC00958	chr11	lincRNA	rs4756736	13014898	5upstream
		FCGRT	chr19	protein_coding	rs2946865	50010378	non-coding intronic
		FCGRT	chr19	protein_coding	rs1132990	50028163	3downstream,3utr,intronic,non-coding
		TRPM4	chr19	protein_coding	rs11882563	49661547	non-coding intronic,intronic,3utr
		TRPM4	chr19	protein_coding	rs11083963	49665340	non-coding intronic,intronic
		TRPM4	chr19	protein_coding	rs73048855	49669561	non-coding intronic,5upstream,intronic

Table S3: Annotations from HaploReg and RegulomeDB database,

		chr	pos (hg38)	variant	Promoter histone marks	Enhancer histone marks	DNAse	Proteins bound	Motifs changed	RegulomeDB Rank
Limbic Region	Amygdala	3	49015786	rs7100		SKIN, BRST	SKIN,SKIN		Irf,SP1,SREBP	4
		14	77423971	rs141260780					CACD,Isl2,Pax-4	7
		14	77423203	rs11846861						5
		14	77457440	rs11845345	24 tissues		46 tissues	9 bound proteins	AP-1	4
		12	56152227	rs3809134	24 tissues		52 tissues		5 altered motifs	4
	Hippocampus	18	9913933	rs4798889	24 tissues		53 tissues	17 bound proteins	BDP1,HEY1,Pax-5	4
		11	86672637	rs670736	22 tissues	7 tissues	26 tissues		4 altered motifs	4
		7	141550885	rs7790742	24 tissues		30 tissues		PU.1	4
		7	141551244	rs7795885	24 tissues		41 tissues	24 bound proteins	AP-2,Rad21,YY1	4
		6	116756287	rs9387433					Nr2e3	5
		6	116765102	rs641338	21 tissues	BLD, GI, SPLN	6 tissues	SUZ12	7 altered motifs	2c
		2	221574191	rs149636195	22 tissues	4 tissues	41 tissues			4
Basal ganglia Region	Accumbens area	3	46877889	rs2168442		11 tissues			5 altered motifs	4
		3	46892800	rs144645644	19 tissues	12 tissues	9 tissues			4
		11	9384582	rs75955853	24 tissues	HRT	53 tissues	42 bound proteins	11 altered motifs	2a
		11	9388254	rs12363308		ESDR, BLD, SKIN			6 altered motifs	4
	Caudate	3	112991286	rs114429530	24 tissues		52 tissues	30 bound proteins	6 altered motifs	2a
		18	62187020	rs3752091	24 tissues		53 tissues	17 bound proteins	8 altered motifs	2b
		18	62191568	rs9958695	ESDR	5 tissues	BLD		ELF1,Mef2,Spz1	7
		16	54286923	rs191251428	22 tissues	9 tissues	38 tissues	POL24H8,HAE2F1	9 altered motifs	2a
		1	16044217	rs75909377		5 tissues			5 altered motifs	4
		12	56334353	rs79824801	24 tissues		53 tissues	12 bound proteins	BCL,Ik-2	4
		4	37686405	rs3832308	24 tissues		53 tissues	12 bound proteins	22 altered motifs	2a
		4	37686821	rs4832933	24 tissues		53 tissues	5 bound proteins	4 altered motifs	2b
		1	25342335	rs3093586		6 tissues			PLZF	7
		1	25342566	rs3091243		6 tissues			E2F,Pou2f2	5
		1	25360817	rs8876		9 tissues	ESDR,LNG,BRN	CEBPB		4
		21	36057947	rs2835263		8 tissues	BLD,BLD		5 altered motifs	7
		21	36079000	rs142847892	BLD, GI	8 tissues		STAT2	PLZF,ZBTB33	5
	Putamen	6	150068788	rs1537648	24 tissues		20 tissues		6 altered motifs	4
		14	21106531	rs10872886			20 tissues	AP2ALPHA,BAF155,BRG1		4

Cerebellum Region	Cerebellum	16	13919809	rs6498486	23 tissues	5 tissues	52 tissues	16 bound proteins	CEBPB,Nanog	2b
		16	13920198	rs3136042	24 tissues		53 tissues	20 bound proteins	Mxi1,SREBP	3a
		16	13920421	rs1799798	24 tissues		40 tissues	POL2		4
		3	149129654	rs13089410	24 tissues		53 tissues	35 bound proteins	4 altered motifs	4
		3	149130286	rs7643410	23 tissues	SPLN	24 tissues		CEBPB,Zfp105	2b
		12	57586758	rs10747780					7 altered motifs	4
		12	57610139	rs10437954			10 tissues		NF-kappaB,Zfp161,Znf143	4
		19	49325598	rs8102658		BLD	BLD,BLD			5
		8	81696643	rs2955002					5 altered motifs	1f
		8	81697548	rs58379275					AP-1,BDP1,Maf	4
		8	81713911	rs75348453					Mef2	5
		6	159760403	rs2025187	4 tissues	18 tissues	ESC,KID	POL2	LUN-1,Osr,Pitx2	3a
		1	52142437	rs627011	24 tissues		20 tissues	4 bound proteins	14 altered motifs	4
		13	102883478	rs1886087					EWSR1-FLI1,HDAC2,Spdef	6
		13	102889266	rs9518861						7
		13	102889870	rs9554903					Foxp3	7
		17	39687428	rs2517955	21 tissues	10 tissues	19 tissues	6 bound proteins	7 altered motifs	2a
		17	39687509	rs75849983	21 tissues	10 tissues	21 tissues	4 bound proteins	LBP-1	4
		11	12989533	rs111880988	6 tissues	ESDR, BLD	13 tissues	27 bound proteins	EWSR1-FLI1,PRDM1,TFII-I	4
		11	12993351	rs4756736		BRST, SKIN, BLD			4 altered motifs	6
		19	49507121	rs2946865	ESC, IPSC, BLD	6 tissues	IPSC,BLD, BLD		AhR,CHOP::CEBPalpha	5
		19	49524906	rs1132990		IPSC, PLCNT, SPLN			Pbx3,Tgif1,ZBTB33	3a
		19	49158290	rs11882563	24 tissues	BLD	23 tissues	5 bound proteins	9 altered motifs	4
		19	49162083	rs11083963		10 tissues	5 tissues	GATA2	21 altered motifs	3a
		19	49166304	rs73048855	5 tissues	9 tissues	ESDR,KID, MUS		HNF4,LXR,VDR	2b
		**Total**		**56**	**32 (57.1%)**	**29 (51.8%)**		**25 (44.6%)**	**49 (87.5%)**	

Table S4: Annotations of promoters of cis-eQTL SNPs,

Region	Structure	Gene	SNP	BP	CHR	PromoterStart	PromoterEnd	Strand	Gene_name	Gene_id	Gene_type
LimbicRegion	Amygdala	NOXRED1	rs141260780	77890314	chr14	77887860	77890860	-	NOXRED1	ENSG00000165555	protein_coding
		NOXRED1	rs11846861	77889546	chr14	77887860	77890860	-	NOXRED1	ENSG00000165555	protein_coding

		AHSA1	rs11845345	77923783	chr14	77922213	77925213	+	AHSA1	ENSG00000100591	protein_coding
		MYL6B	rs3809134	56546011	chr12	56544040	56547040	+	MYL6B	ENSG00000196465	protein_coding
	Hippocampus	VAPA	rs4798889	9913930	chr18	9911999	9914999	+	VAPA	ENSG00000101558	protein_coding
		ME3	rs670736	86383679	chr11	86381678	86384678	-	ME3	ENSG00000151376	protein_coding
		AGK	rs7790742	141250685	chr7	1.41E+08	141252078	+	AGK	ENSG00000006530	protein_coding
		AGK	rs7795885	141251044	chr7	1.41E+08	141252078	+	AGK	ENSG00000006530	protein_coding
		FAM162B	rs641338	117086265	chr6	1.17E+08	117087882	-	FAM162B	ENSG00000183807	protein_coding
		EPHA4	rs149636195	222438911	chr2	2.22E+08	222439922	-	EPHA4	ENSG00000116106	protein_coding
BasalGangliaRegion	AccumbensArea	PTH1R	rs2168442	46919379	chr3	46917211	46920211	+	PTH1R	ENSG00000160801	protein_coding
		IPO7	rs75955853	9406129	chr11	9404199	9407199	+	IPO7	ENSG00000205339	protein_coding
	Caudate	GTPBP8	rs114429530	112710133	chr3	1.13E+08	112710831	+	GTPBP8	ENSG00000163607	protein_coding
		RELCH	rs3752091	59854253	chr18	59852488	59855488	+	RELCH	ENSG00000134444	protein_coding
		IRX3	rs191251428	54320835	chr16	54318699	54321699	-	IRX3	ENSG00000177508	protein_coding
		CLCNKB	rs75909377	16370712	chr1	16368231	16371231	+	CLCNKB	ENSG00000184908	protein_coding
		IL23A	rs79824801	56728137	chr12	56725958	56728958	+	IL23A	ENSG00000110944	protein_coding
		RELL1	rs4832933	37688443	chr4	37685998	37688998	-	RELL1	ENSG00000181826	protein_coding
		SETD4	rs142847892	37451298	chr21	37449687	37452687	-	SETD4	ENSG00000185917	protein_coding
	Putamen	ULBP3	rs1537648	150389924	chr6	1.5E+08	150391257	-	ULBP3	ENSG00000131019	protein_coding
		ERCC4	rs6498486	14013666	chr16	14012014	14015014	+	ERCC4	ENSG00000175595	protein_coding
		ERCC4	rs3136042	14014055	chr16	14012014	14015014	+	ERCC4	ENSG00000175595	protein_coding
		ERCC4	rs1799798	14014278	chr16	14012014	14015014	+	ERCC4	ENSG00000175595	protein_coding
		HPS3	rs13089410	148847441	chr3	1.49E+08	148848425	+	HPS3	ENSG00000163755	protein_coding
		HPS3	rs7643410	148848073	chr3	1.49E+08	148848425	+	HPS3	ENSG00000163755	protein_coding
CerebellumRegion	Cerebellum	SLC6A16	rs8102658	49828855	chr19	49826472	49829472	-	SLC6A16	ENSG00000063127	protein_coding
		ACAT2	rs2025187	160181435	chr6	1.6E+08	160184077	+	ACAT2	ENSG00000120437	protein_coding
		ZFYVE9	rs627011	52608109	chr1	52605766	52608766	+	ZFYVE9	ENSG00000157077	protein_coding
		ERBB2	rs2517955	37843681	chr17	37842167	37845167	+	ERBB2	ENSG00000141736	protein_coding
		ERBB2	rs75849983	37843762	chr17	37842167	37845167	+	ERBB2	ENSG00000141736	protein_coding
		FCGRT	rs2946865	50010378	chr19	50008073	50011073	+	FCGRT	ENSG00000104870	protein_coding
		TRPM4	rs11882563	49661547	chr19	49658998	49661998	+	TRPM4	ENSG00000130529	protein_coding

Table S5: Annotations of super enhancers of cis-eQTL SNPs,

Region	Structure	GENE	SNP	BP	CHR	Enhancer Start	Enhancer End	Rank	Element	Common_SNP	eQTL	Risk_SNP	TFBS	Crisps_cas9_target_sites	Case_value	Control_value	Overlap_gene	Proximal_gene	Closest_gene	Closest_active_gene	Data_sources	Biosample_type	Tissue_type	Biosample_name
LimbicRegion	Amygdala	MYL6B	rs3809134	56546011	chr12	56536882	56556002	937	3	209	11	1	132	1726	25762.29	2124.232	MYL6B,SMARCC2,ESYT1,MYL6	ESYT1,RPL41,PA2G4,SMARCC2,ZC3H10	MYL6B	MYL6B	ENCODE	Tissue	Adrenal gland	adrenal-gland
		MYL6B	rs3809134	56546011	chr12	56537251	56561886	850	2	209	11	1	129	1675	20538.79	1897.287	MYL6B,SMARCC2,ESYT1,MYL6	ESYT1,RPL41,PA2G4,SMARCC2,ZC3H10	MYL6B	MYL6B	ENCODE	Tissue	Peyers patch	Peyers-patch
		MYL6B	rs3809134	56546011	chr12	56536497	56570720	728	2	220	11	1	143	1962	24549.61	3326.778	MYL6B,SMARCC2,ESYT1,MYL6	ESYT1,RPL41,PA2G4,SMARCC2,ZC3H10	MYL6B	MYL6B	NCBI GEO/SRA	Cell line	Umbilical vein	HUVEC_VEGF_4h
		MYL6B	rs3809134	56546011	chr12	56525918	56572407	185	10	486	30	2	363	4242	43634.58	7201.146	MYL6B,SMARCC2,ESYT1,MYL6	ESYT1,RPL41,PA2G4,SMARCC2,RNF41,OBFC2B,ZC3H10	MYL6B	MYL6B	NCBI GEO/SRA	Cell line	Other	90-8TL_DMSO
		MYL6B	rs3809134	56546011	chr12	56536811	56558466	1052	4	228	11	1	185	2182	7503.458	1847.172	MYL6B,SMARCC2,ESYT1,MYL6	ESYT1,RPL41,PA2G4,SMARCC2,ZC3H10	MYL6B	MYL6B	NCBI GEO/SRA	Other	Skin	NHEK_scramble
		MYL6B	rs3809134	56546011	chr12	56536691	56554236	625	3	189	10	0	118	1459	23826.11	2844.045	MYL6B,ESYT1,MYL6	ESYT1,RPL41,PA2G4,SMARCC2,ZC3H10	MYL6B	MYL6B	NCBI GEO/SRA	Cell line	Colon	V456
		MYL6B	rs3809134	56546011	chr12	56538274	56555291	452	3	192	11	1	119	1378	33567.73	2084.583	MYL6B,ESYT1,MYL6	ESYT1,RPL41,PA2G4,SMARCC2,ZC3H10	MYL6B	MYL6B	NCBI GEO/SRA	Cell line	Colon	V481
	Hippocampus	VAPA	rs4798889	9913930	chr18	9912628	9912712 6	1145	1	64	8	0	34	196	10373.74	767.3588	VAPA	TXNDC2,VAPA	VAPA	VAPA	ENCODE	In vitro differentiated cells	Liver	hepatocyte

VAPA	rs4798889	9913930	chr18	9912865	9930080	1025	4	214	32	0	40	196	24722.46	1812.74	VAPA	TXNDC2,VAPA	VAPA	VAPA	ENCODE	Primary cell	Skin	keratinocyte
VAPA	rs4798889	9913930	chr18	9912838	9934342	729	6	271	43	1	45	236	29978.73	2715.955	VAPA	TXNDC2,VAPA	VAPA	VAPA	ENCODE	Tissue	Adrenal gland	adrenal-gland
VAPA	rs4798889	9913930	chr18	9912811	9916932	945	1	60	8	0	34	196	29524.9	535.3179	VAPA	TXNDC2,VAPA	VAPA	VAPA	ENCODE	Tissue	Spleen	spleen
VAPA	rs4798889	9913930	chr18	9912848	9928313	1267	4	195	28	0	40	196	17733.72	1889.823	VAPA	TXNDC2,VAPA	VAPA	VAPA	ENCODE	Tissue	Tibial nerve	tibial-nerve
VAPA	rs4798889	9913930	chr18	9897907	9918195	503	2	281	35	0	38	196	24806.14	2416.301	VAPA	TXNDC2,VAPA	VAPA	VAPA	NCBI GEO/SRA	In vitro differentiated cells	Blood	Monocyte-derived_dendritic_cells
VAPA	rs4798889	9913930	chr18	9904769	9916677	633	2	167	19	0	35	196	8951.244	1271.774	VAPA	TXNDC2,VAPA	VAPA	VAPA	NCBI GEO/SRA	Stem cell	Umbilical cord	umbilical-cord-derived-mesenchymal-stem-cells_Control_cycle_6
VAPA	rs4798889	9913930	chr18	9912948	9916611	448	1	54	8	0	31	196	38814.98	563.0031	VAPA	TXNDC2,VAPA	VAPA	VAPA	NCBI GEO/SRA	Cell line	Pancreatic	L3-6_4SC-202
VAPA	rs4798889	9913930	chr18	9912527	9954020	549	6	568	100	3	80	400	31111.45	3331.888	VAPA	TXNDC2,VAPA	VAPA	VAPA	NCBI GEO/SRA	Cell line	Pancreatic	L3-6_DMSO
VAPA	rs4798889	9913930	chr18	9912941	9916781	613	1	58	8	0	31	196	39058.18	2377.344	VAPA	TXNDC2,VAPA	VAPA	VAPA	NCBI GEO/SRA	Cell line	Peripheral blood	CUTLL1_dimer-mutant
VAPA	rs4798889	9913930	chr18	9867444	9934533	236	8	966	152	7	80	715	84055.81	6212.441	VAPA,TXNDC2	TXNDC2,VAPA	VAPA	VAPA	NCBI GEO/SRA	Cell line	Colon	HCT116_shPAF1
ME3	rs670736	86383679	chr11	86374754	86418816	564	6	585	105	10	104	282	23771.45	5093.567	ME3		ME3	ME3	NCBI GE	Tissue	Pancreas	pancreatic-islets-F

																		O/SRA			
ME3	rs670736	86383679	chr11	86380848	86418226	604	5	498	92	9	94	282	23892.02	4085.415	ME3	ME3	ME3	NCBI GEO/SRA	Stem cell	Embryo	AFG
ME3	rs670736	86383679	chr11	86379792	86409156	286	4	393	63	5	81	282	42991.83	4187.306	ME3	ME3	ME3	NCBI GEO/SRA	Cell line	Mammary Gland	MDA-MB-231_untreat
ME3	rs670736	86383679	chr11	86373737	86388654	542	3	201	33	3	46	282	35007.22	3135.553	ME3	ME3	ME3	NCBI GEO/SRA	Cell line	Colon	V389
ME3	rs670736	86383679	chr11	86372260	86388843	724	2	233	38	3	46	282	25849.58	1771.064	ME3	ME3	ME3	NCBI GEO/SRA	Cell line	Colon	V481
ME3	rs670736	86383679	chr11	86285626	86408981	349	16	1889	380	11	292	282	47553.35	9547.677	ME3	ME3	ME3	NCBI GEO/SRA	Cell line	Neuroblastoma	GIMEN
ME3	rs670736	86383679	chr11	86379552	86408779	229	5	392	62	5	81	282	32576.41	4661.707	ME3	ME3	ME3	NCBI GEO/SRA	Cell line	Kidney	HEK293A_WT-MEF2B_DMSO_6h
EPHA4	rs149636195	2.22E+08	chr2	2.22E+08	2.22E+08	122	1	80	1	0	58	548	3672.372	937.056	EPHA4	EPHA4	EPHA4	ENCODE	In vitro differentiated cells	Embryo	radial-glial-cell
EPHA4	rs149636195	2.22E+08	chr2	2.22E+08	2.22E+08	1130	5	411	19	1	206	563	17086.39	2797.871	EPHA4	EPHA4	EPHA4	NCBI GEO/SRA	Stem cell	Embryo	AFG
EPHA4	rs149636195	2.22E+08	chr2	2.22E+08	2.22E+08	695	3	297	12	1	315	740	9861.816	2225.886	EPHA4	EPHA4	EPHA4	NCBI GEO/SRA	Other	Foreskin	foreskin_D11

Basal GangliaRegion	AccumbensArea	PTH1R	rs21468442	46919379	chr3	46901829	46935645	984	5	354	44	4	377	867	21621.95	8335.644	MYL3,PTH1R	PRSS42	PTH1R	PTH1R	Roadmap	Tissue	Right cardiac atrium	right-cardiac-atrium
		PTH1R	rs21468442	46919379	chr3	46907726	46935841	92	2	297	36	4	317	581	25997.94	5614.566	PTH1R	PRSS42,MYL3	PTH1R	PTH1R	NCBIGEO/SRA	Tissue	Kidney	Patient_x750N
		PTH1R	rs21468442	46919379	chr3	46908194	46942219	397	6	365	45	3	398	1132	21102.31	4535.533	PTH1R	PRSS42,MYL3	PTH1R	PTH1R	NCBIGEO/SRA	Tissue	Skeletal muscle	RMS008
		PTH1R	rs21468442	46919379	chr3	46899091	46936362	625	3	385	52	4	387	1173	30420.59	5344.661	MYL3,PTH1R	PRSS42	PTH1R	PTH1R	NCBIGEO/SRA	Primary cell	Heart	purified cardiomyocyte G296S mutants
		PTH1R	rs144645644	46934290	chr3	46901829	46935645	984	5	354	44	4	377	867	21621.95	8335.644	MYL3,PTH1R	PRSS42	PTH1R	PTH1R	Roadmap	Tissue	Right cardiac atrium	right-cardiac-atrium
		PTH1R	rs144645644	46934290	chr3	46907726	46935841	92	2	297	36	4	317	581	25997.94	5614.566	PTH1R	PRSS42,MYL3	PTH1R	PTH1R	NCBIGEO/SRA	Tissue	Kidney	Patient_x750N
		PTH1R	rs144645644	46934290	chr3	46908194	46942219	397	6	365	45	3	398	1132	21102.31	4535.533	PTH1R	PRSS42,MYL3	PTH1R	PTH1R	NCBIGEO/SRA	Tissue	Skeletal muscle	RMS008
		PTH1R	rs144645644	46934290	chr3	46899091	46936362	625	3	385	52	4	387	1173	30420.59	5344.661	MYL3,PTH1R	PRSS42	PTH1R	PTH1R	NCBIGEO/SRA	Primary cell	Heart	purified cardiomyocyte G296S mutants
		IPO7	rs75955853	9406129	chr11	9328055	9420101	21	12	1482	209	3	66	321	66788.58	9057.326	TMEM41B,IPO7	SNORA23,DENND5A	IPO7	IPO7	NCBIGEO/SRA	Stem cell	Embryo	Embryonic-stem-cells_Naive
		IPO7	rs75955853	9406129	chr11	9382715	9427986	976	6	605	70	3	44	167	26067.04	4518.046	IPO7	SNORA23,TMEM41B	IPO7	IPO7	NCBIGE	Cell line	Lung	IMR-90_proliferating

																				O/SRA			
	IPO7	rs12940363308	9801	chr11	9328055	9420101	21	12	1482	209	3	66	321	66788.58	9057.326	TMEM41B,IPO7	SNORA23,DENND5A	IPO7	IPO7	NCBIGEO/SRA	Stem cell	Embryo	Embryonic-stem-cells_Naive
	IPO7	rs12940363308	9801	chr11	9382715	9421796	976	6	605	70	3	44	167	26067.04	4518.046	IPO7	SNORA23,TMEM41B	IPO7	IPO7	NCBIGEO/SRA	Cell line	Lung	IMR-90_proliferating
Caudate	GTPBP8	rs114429530	1.13E+08	chr3	1.13E+08	1.13E+08	325	6	620	64	9	139	1175	50029.66	9406.027	GTPBP8, CD200R1, C3orf17	GTPBP8,C3orf17	GTPBP8	GTPBP8	Roadmap	Primary cell	Blood	naive thymus-derived CD4-positive, alpha-beta T cell
	GTPBP8	rs114429530	1.13E+08	chr3	1.13E+08	1.13E+08	407	4	623	66	9	139	1175	32289.29	7050.322	GTPBP8, CD200R1, C3orf17	GTPBP8,C3orf17	GTPBP8	GTPBP8	Roadmap	Primary cell	Blood	CD4-positive-alpha-beta-memory-T-cell
	GTPBP8	rs114429530	1.13E+08	chr3	1.13E+08	1.13E+08	416	5	611	64	9	138	1175	32642.49	5651.964	GTPBP8, CD200R1, C3orf17	GTPBP8,C3orf17	GTPBP8	GTPBP8	Roadmap	Primary cell	Blood	CD8-positive-alpha-beta-T-cell
	GTPBP8	rs114429530	1.13E+08	chr3	1.13E+08	1.13E+08	553	6	607	64	9	138	1175	37772.96	5378.443	GTPBP8, CD200R1, C3orf17	GTPBP8,C3orf17	GTPBP8	GTPBP8	Roadmap	Primary cell	Blood	natural-killer-cell
	GTPBP8	rs114429530	1.13E+08	chr3	1.13E+08	1.13E+08	338	6	666	70	9	148	1175	35100.92	5709.347	GTPBP8, CD200R1, C3orf17	GTPBP8,C3orf17	GTPBP8	GTPBP8	Roadmap	Primary cell	Cord blood	T-cell
	GTPBP8	rs114429530	1.13E+08	chr3	1.13E+08	1.13E+08	671	1	15	3	1	10	128	18391.66	231.2352	GTPBP8	CD200R1,GTPBP8,C3orf17	GTPBP8	GTPBP8	NCBIGEO/SRA	Cell line	Embryo endodermal	CyT49_ES_D10
	IRX3	rs191251428	54320835	chr16	54311719	54322448	507	2	140	7	1	132	773	13913.37	1197.356	IRX3		IRX3	IRX3	ENCODE	In vitro differentiated cells	Embryo	cardiac-muscle-cell
	IRX3	rs191251428	54320835	chr16	54310774	54322453	491	1	161	8	1	132	773	29961.31	944.8311	IRX3		IRX3	IRX3	ENCODE	Tissue	Breast epithelium	breast-epithelium

IRX3	rs191251428	54320835	chr16	54310769	54322404	368	2	160	8	1	132	773	29961.29	4605.133	IRX3	IRX3	IRX3	NCBI GEO/SRA	Cell line	Mammary Gland	MDA-MB-468
IRX3	rs191251428	54320835	chr16	54310284	54321985	238	1	161	8	1	132	773	14358.3	2515.715	IRX3	IRX3	IRX3	NCBI GEO/SRA	Tissue	Kidney	Patient_x750T
IRX3	rs191251428	54320835	chr16	54309775	54342603	186	5	446	18	1	191	773	45036.73	12254.69	IRX3	IRX3	IRX3	NCBI GEO/SRA	Cell line	Haematopoietic and lymphoid tissue	P12-ICHIKAWA
IRX3	rs191251428	54320835	chr16	54311729	54322028	168	2	132	7	1	132	773	45497.89	1006.212	IRX3	IRX3	IRX3	NCBI GEO/SRA	Cell line	Mammary Gland	MCF-7_estradiol
IRX3	rs191251428	54320835	chr16	54298759	54321885	537	3	318	12	1	140	773	35091.39	2654.865	IRX3	IRX3	IRX3	NCBI GEO/SRA	Cell line	Peripheral blood	MV411
IRX3	rs191251428	54320835	chr16	54315325	54326091	227	2	121	1	1	175	773	54961.51	3110.297	IRX3	IRX3	IRX3	NCBI GEO/SRA	Cell line	Pancreatic	L3-6_4SC-202
IRX3	rs191251428	54320835	chr16	54301664	54321971	370	2	282	11	1	139	773	16956.35	2000.24	IRX3	IRX3	IRX3	NCBI GEO/SRA	Cell line	Haematopoietic and lymphoid tissue	MOLM14_untreated
IRX3	rs191251428	54320835	chr16	54268574	54322008	197	7	818	92	1	235	773	47337.18	5872.397	IRX3	IRX3	IRX3	NCBI GEO/SRA	Cell line	Haematopoietic and lymphoid tissue	MOLM14_DMSO_9
IRX3	rs191251428	54320835	chr16	54301411	54321856	252	3	283	11	1	140	773	43928.13	2089.479	IRX3	IRX3	IRX3	NCBI GEO/SRA	Cell line	Haematopoietic and lymphoid tissue	MOLM14_DMSO_10

IRX3	rs191251428	54320835	chr16	54268319	54322229	133	8	828	92	1	236	773	60578.67	6215.823	IRX3		IRX3	IRX3	NCBI GEO/SRA	Cell line	Haematopoietic and lymphoid tissue	MOLM14_CA25_11
IRX3	rs191251428	54320835	chr16	54299532	54322155	158	2	312	11	1	140	773	55976.09	3477.155	IRX3		IRX3	IRX3	NCBI GEO/SRA	Cell line	Haematopoietic and lymphoid tissue	MOLM14_CA25_12
IRX3	rs191251428	54320835	chr16	54301624	54321891	561	2	281	11	1	139	773	31355.08	2136.142	IRX3		IRX3	IRX3	NCBI GEO/SRA	Cell line	Haematopoietic and lymphoid tissue	MV411_untreat
IRX3	rs191251428	54320835	chr16	54311637	54321954	301	2	133	7	1	132	773	22020.6	775.8384	IRX3		IRX3	IRX3	NCBI GEO/SRA	Cell line	Mammary Gland	ZR-75-1_2
IRX3	rs191251428	54320835	chr16	54315346	54321935	644	2	77	1	1	131	773	22660.23	465.8423	IRX3		IRX3	IRX3	NCBI GEO/SRA	Cell line	Pectoralis major muscle	LHCN-M2_nodox
CLCNKB	rs75909377	16370712	chr1	16335059	16422687	63	20	1652	480	8	423	7042	75325.03	4214.907	CLCNKB, HSPB7,FAM131C, CLCNKA	ZBTB17,C1orf64	CLCNKB	CLCNKB	NCBI GEO/SRA	Tissue	Pancreas	pancreatic-islets-M
CLCNKB	rs75909377	16370712	chr1	16338125	16405188	71	6	1335	415	8	370	7042	48795.04	16209.13	CLCNKB, HSPB7,FAM131C, CLCNKA	ZBTB17,C1orf64	CLCNKB	CLCNKB	NCBI GEO/SRA	Cell line	Pancreatic endoderm	IIDP/donor790
CLCNKB	rs75909377	16370712	chr1	16350781	16417853	27	20	1313	366	5	330	5297	29605.58	3682.253	CLCNKB,FAM131C,CLCNKA	ZBTB17,C1orf64,HSPB7	CLCNKB	CLCNKB	NCBI GEO/SRA	Other	Skin	NHEK_scramble
IL23A	rs79824801	56728137	chr12	56726404	56734201	873	2	50	9	0	126	611	28034.89	1008.932	IL23A,PAN2	CNPY2,STAT2,APOF,PAN2,CS	IL23A	IL23A	Roadmap	Primary cell	Blood	CD14-positive-monocyte
IL23A	rs79824801	56728137	chr12	56726311	56735941	360	2	66	11	0	130	733	40421.93	2047.338	IL23A,STAT2,PAN2	CNPY2,APOF,PAN2,CS	IL23A	IL23A	Roadmap	Primary cell	Blood	naive thymus-derived CD4-

																						positive, alpha-beta T cell
IL23A	rs79824801	56728137	chr12	56726184	56736116	369	2	73	12	0	130	757	27662.61	1559.324	IL23A,STAT2,PAN2	CNPY2,APOF,PAN2,CS	IL23A	IL23A	Roadmap	Primary cell	Blood	CD4-positive-alpha-beta-memory-T-cell
IL23A	rs79824801	56728137	chr12	56726171	56734638	441	2	60	11	0	126	631	27441.55	1004.186	IL23A,PAN2	CNPY2,STAT2,APOF,PAN2,CS	IL23A	IL23A	Roadmap	Primary cell	Blood	CD8-positive-alpha-beta-T-cell
IL23A	rs79824801	56728137	chr12	56726215	56735828	370	2	68	11	0	130	717	29132.2	1046.856	IL23A,STAT2,PAN2	CNPY2,APOF,PAN2,CS	IL23A	IL23A	Roadmap	Primary cell	Cord blood	T-cell
IL23A	rs79824801	56728137	chr12	56726469	56735687	602	5	61	9	0	130	698	14548.77	999.2312	IL23A,STAT2,PAN2	CNPY2,APOF,PAN2,CS	IL23A	NA	NCBI GEO/SRA	Primary cell	Blood	CD8donorA
IL23A	rs79824801	56728137	chr12	56726267	56734274	926	1	55	11	0	126	618	23542.98	988.8645	IL23A,PAN2	CNPY2,STAT2,APOF,PAN2,CS	IL23A	IL23A	NCBI GEO/SRA	Cell line	Peripheral blood	MV411
RELL1	rs4832933	37688443	chr4	37648645	37702520	905	9	781	37	6	104	291	30757.24	7720.288	RELL1		RELL1	RELL1	Roadmap	Tissue	Large intestine	large-intestine_108d
RELL1	rs4832933	37688443	chr4	37666273	37702406	1277	6	505	16	1	77	191	22221.79	4682.837	RELL1		RELL1	RELL1	Roadmap	Tissue	Small intestine	small-intestine_108days
RELL1	rs4832933	37688443	chr4	37663045	37702602	835	9	548	17	1	83	191	12231.02	3310.921	RELL1		RELL1	RELL1	Roadmap	Tissue	Stomach	stomach_3y
RELL1	rs4832933	37688443	chr4	37633283	37704765	550	8	1047	61	9	128	422	37435.12	7284.016	RELL1	C4orf19	RELL1	RELL1	ENCODE	Tissue	Stomach	stomach
TMEM50A	rs3093586	25668826	chr1	25634776	25710646	207	14	815	116	2	31	1148	76044.5	8338.113	RHD,TMEM50A,RHCE	RHD,TMEM57,RHCE	TMEM50A	TMEM50A	ENCODE	Cell line	Submandibular Salivary Gland	ACC112
TMEM50A	rs3093586	25668826	chr1	25634404	25671268	495	5	412	74	0	18	741	29218.41	7612.416	RHD,TMEM50A	RHD	TMEM50A	TMEM50A	NCBI GEO/SRA	Cell line	Endometrium	EEC16
TME	rs3093586	25668826	chr1	25634922	25710617	1052	12	808	113	2	31	1148	31655.65	11142.3	RHD,TMEM50A,RHCE	RHD,TMEM57,RHCE	TMEM50A	TMEM50A	NCBIGE	Cell line	Lung	IMR-90_proliferating

M50A																	O/S RA			
TMEM50A	rs3093586	25668826	chr1	256256 420709270 59 65	4	317	42	0	18	729	45087.58	3965.903	RHD,TMEM50A	RHD	TMEM50A	TMEM50A	NCBI GEO/SRA	Cell line	Urinary bladder	T24
TMEM50A	rs3093586	25668826	chr1	256256 441905419 08 17	7	512	55	1	23	930	18433.65	3740.565	RHD,TMEM50A,RHCE	RHD	TMEM50A	TMEM50A	NCBI GEO/SRA	Primary cell	Tonsil	Th1
TMEM50A	rs3093586	25668826	chr1	256256 526917695 96 36	7	408	41	1	22	889	20609.22	3673.664	RHD,TMEM50A,RHCE		TMEM50A	TMEM50A	NCBI GEO/SRA	Primary cell	Tonsil	Th17
TMEM50A	rs3093586	25668826	chr1	256256 332716478 49 92	4	436	80	0	18	849	14489.17	5401.242	RHD,TMEM50A	RHD	TMEM50A	TMEM50A	NCBI GEO/SRA	Other	Foreskin	foreskin_HFFs
TMEM50A	rs3093586	25668826	chr1	256257 336118411 12 09	9	843	124	2	31	1209	40248	12863.41	RHD,TMEM50A,RHCE	RHD,TMEM57,RHCE	TMEM50A	TMEM50A	NCBI GEO/SRA	Cell line	Osteoblastic	hFOB_differentiated
TMEM50A	rs3093586	25668826	chr1	256257 346107350 92 30	10	815	116	2	31	1148	42862.62	11071.13	RHD,TMEM50A,RHCE	RHD,TMEM57,RHCE	TMEM50A	TMEM50A	NCBI GEO/SRA	Cell line	Osteoblastic	hFOB_JQ1_differentiated
TMEM50A	rs3093586	25668826	chr1	256257 344107184 82 90	12	818	117	2	31	1150	55018.07	11392.78	RHD,TMEM50A,RHCE	RHD,TMEM57,RHCE	TMEM50A	TMEM50A	NCBI GEO/SRA	Cell line	Osteoblastic	hFOB_undifferentiated
TMEM50A	rs3093586	25668826	chr1	256256 346735349 93 88	5	448	74	0	18	729	64359.56	15060.14	RHD,TMEM50A	RHD	TMEM50A	TMEM50A	NCBI GEO/SRA	Cell line	Skeletal muscle	7250-PAX3-FOXO1
TME	rs3093586	25668826	chr1	256257 450005214 62 76	11	572	60	2	25	972	49096.58	5024.017	RHD,TMEM50A,RHCE	RHD,RHCE	TMEM50A	TMEM50A	NCBI GE	Tissue	Pelvis	patient 119

M50A																		O/S RA			
TMEM50A	rs3093586	25668826	chr1	25646893	25670450	517	6	248	31	0	15	641	9785.578	1849.225	RHD,TMEM50A	RHD	TMEM50A	TMEM50A	NCBIGEO/SRA	Cell line	Pectoralis major muscle	LHCN-M2_dox_6h
TMEM50A	rs3091243	25669057	chr1	25634776	25710646	207	14	815	116	2	31	1148	76044.5	8338.113	RHD,TMEM50A,RHCE	RHD,TMEM57,RHCE	TMEM50A	TMEM50A	ENCODE	Cell line	Submandibular Salivary Gland	ACC112
TMEM50A	rs3091243	25669057	chr1	25634404	25671268	495	5	412	74	0	18	741	29218.41	7612.416	RHD,TMEM50A	RHD	TMEM50A	TMEM50A	NCBIGEO/SRA	Cell line	Endometrium	EEC16
TMEM50A	rs3091243	25669057	chr1	25634922	25710617	1052	12	808	113	2	31	1148	31655.65	11142.3	RHD,TMEM50A,RHCE	RHD,TMEM57,RHCE	TMEM50A	TMEM50A	NCBIGEO/SRA	Cell line	Lung	IMR-90_proliferating
TMEM50A	rs3091243	25669057	chr1	25642059	25670965	270	4	317	42	0	18	729	45087.58	3965.903	RHD,TMEM50A	RHD	TMEM50A	TMEM50A	NCBIGEO/SRA	Cell line	Urinary bladder	T24
TMEM50A	rs3091243	25669057	chr1	25644108	25690517	419	7	512	55	1	23	930	18433.65	3740.565	RHD,TMEM50A,RHCE	RHD	TMEM50A	TMEM50A	NCBIGEO/SRA	Primary cell	Tonsil	Th1
TMEM50A	rs3091243	25669057	chr1	25652696	25691736	695	7	408	41	1	22	889	20609.22	3673.664	RHD,TMEM50A,RHCE		TMEM50A	TMEM50A	NCBIGEO/SRA	Primary cell	Tonsil	Th17
TMEM50A	rs3091243	25669057	chr1	25633249	25671692	478	4	436	80	0	18	849	14489.17	5401.242	RHD,TMEM50A	RHD	TMEM50A	TMEM50A	NCBIGEO/SRA	Other	Foreskin	foreskin_HFFs
TME	rs3091243	25669057	chr1	25633612	25711809	411	9	843	124	2	31	1209	40248	12863.41	RHD,TMEM50A,RHCE	RHD,TMEM57,RHCE	TMEM50A	TMEM50A	NCBIGE	Cell line	Osteoblastic	hFOB_differentiated

M50A																		O/SRA			
TMEM50A	rs3091243	25669057	chr1	25634692	25710730	350	10	815	116	2	31	1148	42862.62	11071.13	RHD,TMEM50A,RHCE	RHD,TMEM57,RHCE	TMEM50A	TMEM50A	NCBI GEO/SRA	Cell line	Osteoblastic	hFOB_JQ1_differentiated
TMEM50A	rs3091243	25669057	chr1	25634482	25710790	184	12	818	117	2	31	1150	55018.07	11392.78	RHD,TMEM50A,RHCE	RHD,TMEM57,RHCE	TMEM50A	TMEM50A	NCBI GEO/SRA	Cell line	Osteoblastic	hFOB_undifferentiated
TMEM50A	rs3091243	25669057	chr1	25634693	25673588	349	5	448	74	0	18	729	64359.56	15060.14	RHD,TMEM50A	RHD	TMEM50A	TMEM50A	NCBI GEO/SRA	Cell line	Skeletal muscle	7250-PAX3-FOXO1
TMEM50A	rs3091243	25669057	chr1	25645062	25700076	214	11	572	60	2	25	972	49096.58	5024.017	RHD,TMEM50A,RHCE	RHD,RHCE	TMEM50A	TMEM50A	NCBI GEO/SRA	Tissue	Pelvis	patient 119
TMEM50A	rs3091243	25669057	chr1	25646893	25670450	517	6	248	31	0	15	641	9785.578	1849.225	RHD,TMEM50A	RHD	TMEM50A	TMEM50A	NCBI GEO/SRA	Cell line	Pectoralis major muscle	LHCN-M2_dox_6h
TMEM50A	rs8876	25687308	chr1	25634776	25710646	207	14	815	116	2	31	1148	76044.5	8338.113	RHD,TMEM50A,RHCE	RHD,TMEM57,RHCE	TMEM50A	TMEM50A	ENCODE	Cell line	Submandibular Salivary Gland	ACC112
TMEM50A	rs8876	25687308	chr1	25634922	25710617	1052	12	808	113	2	31	1148	31655.65	11142.3	RHD,TMEM50A,RHCE	RHD,TMEM57,RHCE	TMEM50A	TMEM50A	NCBI GEO/SRA	Cell line	Lung	IMR-90_proliferating
TMEM50A	rs8876	25687308	chr1	25644108	25690517	419	7	512	55	1	23	930	18433.65	3740.565	RHD,TMEM50A,RHCE	RHD	TMEM50A	TMEM50A	NCBI GEO/SRA	Primary cell	Tonsil	Th1
TME	rs8876	25687308	chr1	25652696	25691736	695	7	408	41	1	22	889	20609.22	3673.664	RHD,TMEM50A,RHCE		TMEM50A	TMEM50A	NCBI GE	Primary cell	Tonsil	Th17

		M50A																			O/SRA			
		TMEM50A	rs8876	25687308	chr1	25633612	25711809	411	9	843	124	2	31	1209	40248	12863.41	RHD,TMEM50A,RHCE	RHD,TMEM57,RHCE	TMEM50A	TMEM50A	NCBI GEO/SRA	Cell line	Osteoblastic	hFOB_differentiated
		TMEM50A	rs8876	25687308	chr1	25634692	25710730	350	10	815	116	2	31	1148	42862.62	11071.13	RHD,TMEM50A,RHCE	RHD,TMEM57,RHCE	TMEM50A	TMEM50A	NCBI GEO/SRA	Cell line	Osteoblastic	hFOB_JQ1_differentiated
		TMEM50A	rs8876	25687308	chr1	25634482	25710790	184	12	818	117	2	31	1150	55018.07	11392.78	RHD,TMEM50A,RHCE	RHD,TMEM57,RHCE	TMEM50A	TMEM50A	NCBI GEO/SRA	Cell line	Osteoblastic	hFOB_undifferentiated
		TMEM50A	rs8876	25687308	chr1	25645062	25700576	214	11	572	60	2	25	972	49096.58	5024.017	RHD,TMEM50A,RHCE	RHD,RHCE	TMEM50A	TMEM50A	NCBI GEO/SRA	Tissue	Pelvis	patient 119
Cerebellum Region	Cerebellum	ACAT2	rs2025187	1.6E+08	chr6	1.6E+08	1.6E+08	1209	2	83	21	1	13	545	10529.43	1000.79	ACAT2,LOC100129518	MRPL18,PNLDC1,SNORA20,WTAP,SNORA29,TCP1	ACAT2	ACAT2	Roadmap	In vitro differentiated cells	Embryo	mesendoderm
		ACAT2	rs2025187	1.6E+08	chr6	1.6E+08	1.6E+08	1475	3	118	25	1	65	671	12561.77	765.6582	ACAT2, WTAP,LOC100129518	MRPL18,PNLDC1,SNORA20,WTAP,SNORA29,TCP1	ACAT2	ACAT2	NCBI GEO/SRA	Stem cell	Embryo	DE
		ZFYVE9	rs627011	52608109	chr1	52604518	52609457	790	1	42	9	0	60	210	27470.72	374.8701	ZFYVE9	ZFYVE9	ZFYVE9	ZFYVE9	ENCODE	Primary cell	Skin	keratinocyte
		ZFYVE9	rs627011	52608109	chr1	52604764	52609629	958	1	43	9	0	60	210	20640.74	591.0975	ZFYVE9	ZFYVE9	ZFYVE9	ZFYVE9	ENCODE	Primary cell	Mammary Gland	mammary-epithelial-cell
		ZFYVE9	rs627011	52608109	chr1	52592947	52609247	747	2	180	28	0	69	210	6627.58	881.83	ZFYVE9	ZFYVE9	ZFYVE9	ZFYVE9	NCBI GEO/SRA	Stem cell	Foreskin	Epidermal-Stem-Cells_shCTRL
		ZFYVE9	rs627011	52608109	chr1	52592848	52609078	401	2	178	28	0	69	210	12732.43	2528.634	ZFYVE9	ZFYVE9	ZFYVE9	ZFYVE9	GGR	Primary cell	Foreskin	foreskin-keratinocyte-calcium5.5day

ERBB2	rs2517955	37843681	chr17	37843440	37847641	546	1	37	5	2	1	211	13482.27	379.3503	ERBB2,PGAP3	C17orf37,GRB7,PNMT,ERBB2,TCAP	ERBB2	ERBB2	NCBI GEO/SRA	Stem cell	Embryo	H7
ERBB2	rs2517955	37843681	chr17	37843418	37847380	413	1	35	5	2	1	211	9587.248	746.837	ERBB2,PGAP3	C17orf37,GRB7,PNMT,ERBB2,TCAP	ERBB2	ERBB2	NCBI GEO/SRA	Cell line	Embryo endodermal	CyT49_ES_D0
ERBB2	rs2517955	37843681	chr17	37842667	37869490	1	2	244	26	6	142	1358	464821.1	119220.2	ERBB2,PGAP3	STARD3,C17orf37,GRB7,PNMT,TCAP	ERBB2	ERBB2	NCBI GEO/SRA	Cell line	Lung	Calu-3
ERBB2	rs2517955	37843681	chr17	37843203	37862327	672	3	186	21	4	99	633	8921.346	1474.46	ERBB2,PGAP3	STARD3,C17orf37,GRB7,PNMT,TCAP	ERBB2	ERBB2	NCBI GEO/SRA	Stem cell	Embryo	Embryonic-stem-cells_Primed
ERBB2	rs2517955	37843681	chr17	37828149	37863777	141	4	344	72	10	117	1581	33525.95	3131.701	ERBB2,PGAP3	PPP1R1B,STARD3,GRB7,C17orf37,PNMT,TCAP	ERBB2	ERBB2	NCBI GEO/SRA	Cell line	Mammary Gland	ZR-75-1_2
ERBB2	rs75849983	37843762	chr17	37843440	37847641	546	1	37	5	2	1	211	13482.27	379.3503	ERBB2,PGAP3	C17orf37,GRB7,PNMT,ERBB2,TCAP	ERBB2	ERBB2	NCBI GEO/SRA	Stem cell	Embryo	H7
ERBB2	rs75849983	37843762	chr17	37843418	37847380	413	1	35	5	2	1	211	9587.248	746.837	ERBB2,PGAP3	C17orf37,GRB7,PNMT,ERBB2,TCAP	ERBB2	ERBB2	NCBI GEO/SRA	Cell line	Embryo endodermal	CyT49_ES_D0
ERBB2	rs75849983	37843762	chr17	37842667	37869490	1	2	244	26	6	142	1358	464821.1	119220.2	ERBB2,PGAP3	STARD3,C17orf37,GRB7,PNMT,TCAP	ERBB2	ERBB2	NCBI GEO/SRA	Cell line	Lung	Calu-3
ERBB2	rs75849983	37843762	chr17	37843203	37862327	672	3	186	21	4	99	633	8921.346	1474.46	ERBB2,PGAP3	STARD3,C17orf37,GRB7,PNMT,TCAP	ERBB2	ERBB2	NCBI GEO/SRA	Stem cell	Embryo	Embryonic-stem-cells_Primed

ERBB2	rs75849983	37843762	chr17	37828149	37863777	141	4	344	72	10	117	1581	33525.95	3131.701	ERBB2,PGAP3	PPP1R1B,STARD3,GRB7,C17orf37,PNMT,TCAP	ERBB2	ERBB2	NCBI GEO/SRA	Cell line	Mammary Gland	ZR-75-1_2
FCGRT	rs2946865	50010378	chr19	50002167	50032071	901	4	427	70	2	74	1719	26713.24	2861.813	FCGRT,RPS11,MIR150,RCN3	RPL13AP5,SNORD33,SNORD34,SNORD32A,ALDH16A1,PIH1D1,SNORD35A,FLT3LG,SNORD35B	FCGRT	FCGRT	Roadmap	Tissue	Spleen	spleen_30y
FCGRT	rs2946865	50010378	chr19	50009969	50020833	179	1	141	27	1	23	733	33506.75	808.2816	FCGRT	RPL13AP5,SNORD33,SNORD34,SNORD32A,RPS11,RCN3,SNORD35A,FLT3LG,MIR150,SNORD35B	FCGRT	FCGRT	ENCODE	Cell line	Brain: derived from metastatic site: supra-orbital area	SK-N-MC
FCGRT	rs1132990	50028163	chr19	50002167	50032071	901	4	427	70	2	74	1719	26713.24	2861.813	FCGRT,RPS11,MIR150,RCN3	RPL13AP5,SNORD33,SNORD34,SNORD32A,ALDH16A1,PIH1D1,SNORD35A,FLT3LG,SNORD35B	FCGRT	FCGRT	Roadmap	Tissue	Spleen	spleen_30y
TRPM4	rs11882563	49661547	chr19	49651032	49670398	451	2	313	41	0	114	2028	29560.26	4527.771	HRC,TRPM4,PPFIA3	LIN7B,C19orf73,TRPM4	TRPM4	TRPM4	Roadmap	Tissue	Lung	lung_3y
TRPM4	rs11882563	49661547	chr19	49651589	49671199	550	3	332	43	0	105	1980	9181.402	1996.298	HRC,TRPM4,PPFIA3	LIN7B,C19orf73,TRPM4	TRPM4	TRPM4	ENCODE	Tissue	Prostate	prostate
TRPM4	rs11882563	49661547	chr19	49651820	49669648	255	3	299	39	0	105	1895	14799.02	2429.956	HRC,TRPM4,PPFIA3	LIN7B,C19orf73,TRPM4	TRPM4	TRPM4	NCBI GEO/SRA	Cell line	Prostate	C4-2B_vehicle_24h
TRPM4	rs11882563	49661547	chr19	49651404	49674733	292	4	385	50	0	142	2322	42393.46	501.5735	HRC,TRPM4,PPFIA3	LIN7B,C19orf73,TRPM4	TRPM4	TRPM4	NCBI GEO/SRA	Tissue	Endometrioid	endometrioid_adenocarcinoma_TumorA
TRPM4	rs11882563	49661547	chr19	49652696	49672329	431	2	342	46	0	129	1968	32838.16	1829.796	HRC,TRPM4,PPFIA3	LIN7B,C19orf73,TRPM4	TRPM4	TRPM4	NCBI GEO/SRA	Primary cell	Heart	purified cardiomyocyte WT
TRPM4	rs11882563	49661547	chr19	49650975	49672559	505	3	362	50	0	147	2313	30657.91	2233.944	HRC,TRPM4,PPFIA3	LIN7B,C19orf73,TRPM4	TRPM4	TRPM4	NCBI GEO/SRA	Primary cell	Heart	purified cardiomyocyte G296S mutants
TRPM4	rs11083963	49665340	chr19	49651032	49670398	451	2	313	41	0	114	2028	29560.26	4527.771	HRC,TRPM4,PPFIA3	LIN7B,C19orf73,TRPM4	TRPM4	TRPM4	Roadmap	Tissue	Lung	lung_3y

TRPM4	rs11083963	49665340	chr19	49651589	49671199	550	3	332	43	0	105	1980	9181.402	1996.298	HRC,TRPM4,PPFIA3	LIN7B,C19orf73,TRPM4	TRPM4	TRPM4	ENCODE	Tissue	Prostate	prostate
TRPM4	rs11083963	49665340	chr19	49651820	49669648	255	3	299	39	0	105	1895	14799.02	2429.956	HRC,TRPM4,PPFIA3	LIN7B,C19orf73,TRPM4	TRPM4	TRPM4	NCBI GEO/SRA	Cell line	Prostate	C4-2B_vehicle_24h
TRPM4	rs11083963	49665340	chr19	49651404	49674733	292	4	385	50	0	142	2322	42393.46	501.5735	HRC,TRPM4,PPFIA3	LIN7B,C19orf73,TRPM4	TRPM4	TRPM4	NCBI GEO/SRA	Tissue	Endometrioid	endometrioid_adenocarcinoma_TumorA
TRPM4	rs11083963	49665340	chr19	49652696	49672329	431	2	342	46	0	129	1968	32838.16	1829.796	HRC,TRPM4,PPFIA3	LIN7B,C19orf73,TRPM4	TRPM4	TRPM4	NCBI GEO/SRA	Primary cell	Heart	purified cardiomyocyte WT
TRPM4	rs11083963	49665340	chr19	49650975	49672559	505	3	362	50	0	147	2313	30657.91	2233.944	HRC,TRPM4,PPFIA3	LIN7B,C19orf73,TRPM4	TRPM4	TRPM4	NCBI GEO/SRA	Primary cell	Heart	purified cardiomyocyte G296S mutants
TRPM4	rs73048855	49669561	chr19	49651032	49670398	451	2	313	41	0	114	2028	29560.26	4527.771	HRC,TRPM4,PPFIA3	LIN7B,C19orf73,TRPM4	TRPM4	TRPM4	Roadmap	Tissue	Lung	lung_3y
TRPM4	rs73048855	49669561	chr19	49651589	49671199	550	3	332	43	0	105	1980	9181.402	1996.298	HRC,TRPM4,PPFIA3	LIN7B,C19orf73,TRPM4	TRPM4	TRPM4	ENCODE	Tissue	Prostate	prostate
TRPM4	rs73048855	49669561	chr19	49651820	49669648	255	3	299	39	0	105	1895	14799.02	2429.956	HRC,TRPM4,PPFIA3	LIN7B,C19orf73,TRPM4	TRPM4	TRPM4	NCBI GEO/SRA	Cell line	Prostate	C4-2B_vehicle_24h
TRPM4	rs73048855	49669561	chr19	49651404	49674733	292	4	385	50	0	142	2322	42393.46	501.5735	HRC,TRPM4,PPFIA3	LIN7B,C19orf73,TRPM4	TRPM4	TRPM4	NCBI GEO/SRA	Tissue	Endometrioid	endometrioid_adenocarcinoma_TumorA
TRPM4	rs73048855	49669561	chr19	49652696	49672329	431	2	342	46	0	129	1968	32838.16	1829.796	HRC,TRPM4,PPFIA3	LIN7B,C19orf73,TRPM4	TRPM4	TRPM4	NCBI GEO/SRA	Primary cell	Heart	purified cardiomyocyte WT

TRPM4	rs73048855	49669561	chr19	49650975	49672559	505	3	362	50	0	147	2313	30657.91	2233.944	HRC,TRPM4,PPFIA3	LIN7B,C19orf73,TRPM4	TRPM4	TRPM4	NCBI GEO/SRA	Primary cell	Heart	purified cardiomyocyte G296S mutants

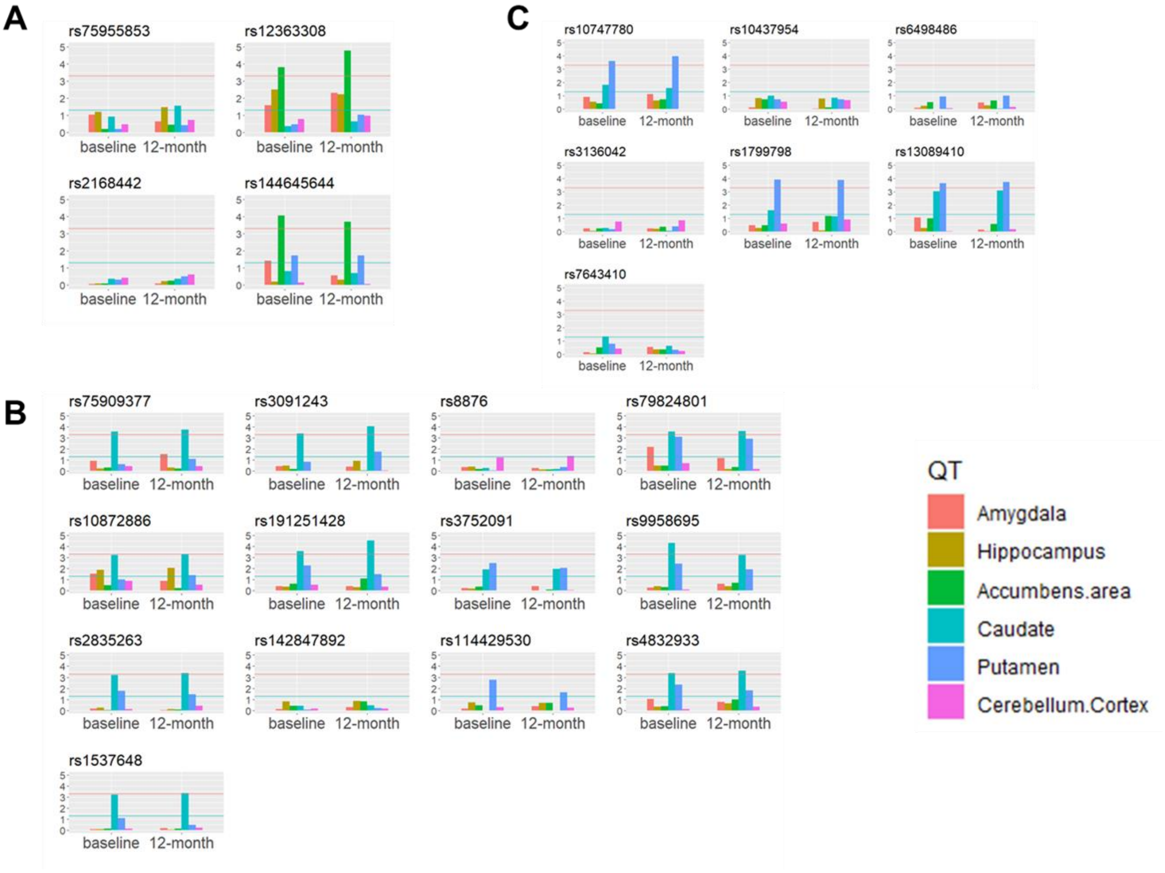

Figure S1. Bar plots of associations between 26 SNPs in basal ganglia region and 6 subcortical structures. (A) 4 SNPs derived from previous step in accumbens area. (B) 13 SNPs derived from previous step in caudate. (C) 7 SNPs derived from previous step in putamen. X-axial presents six subcortical structures (amygdala, hippocampus, accumbens area, caudate, putamen and cerebellum cortex) at baseline and 12-month`s follow-up. Y-axial presents the p-value (-log10) of association based on QT-GWAS. Blue horizontal line represents -log10(0.05) while red horizontal line represents -log10(5e-4).

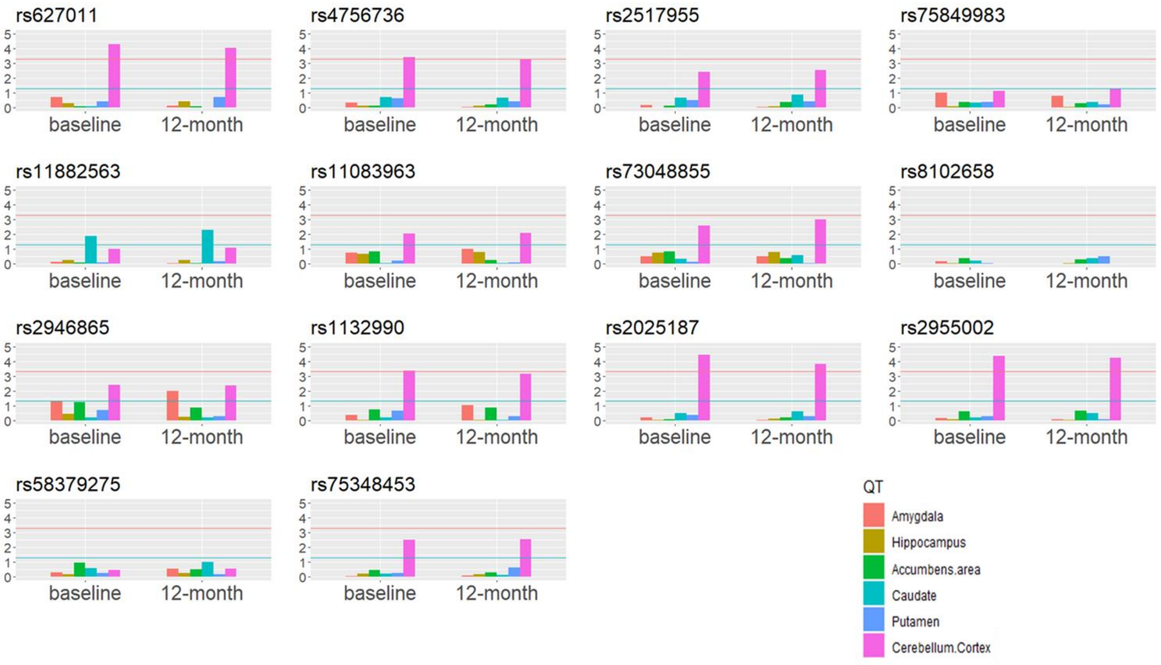

Figure S2. Bar plots of associations between 14 SNPs in cerebellum region and 6 subcortical structures. X-axial presents six subcortical structures (amygdala, hippocampus, accumbens area, caudate, putamen and cerebellum cortex) at baseline and 12-month`s follow-up. Y-axial presents the p-value (-log10) of association based on QT-GWAS. Blue horizontal line represents -log10(0.05) while red horizontal line represents -log10(5e-4).
